# Long circulating tracer tailored for magnetic particle imaging

**DOI:** 10.7150/ntno.58548

**Published:** 2021-03-24

**Authors:** Sitong Liu, Andreina Chiu-Lam, Angelie Rivera-Rodriguez, Ryan DeGroff, Shehaab Savliwala, Nicole Sarna, Carlos M. Rinaldi-Ramos

**Affiliations:** 1 Department of Chemical Engineering, University of Florida, Gainesville, FL 32611, USA.; 2 J. Crayton Pruitt Family Department of Biomedical Engineering, University of Florida, Gainesville, FL 32611-6131, USA.

**Keywords:** magnetic particle imaging, iron oxide nanoparticles, long circulating tracer

## Abstract

Superparamagnetic iron oxide nanoparticle (SPION) tracers possessing long blood circulation time and tailored for magnetic particle imaging (MPI) performance are crucial for the development of this emerging molecular imaging modality. Here, single-core SPION MPI tracers coated with covalently bonded polyethyelene glycol (PEG) brushes were obtained using a semi-batch thermal decomposition synthesis with controlled addition of molecular oxygen, followed by an optimized PEG-silane ligand exchange procedure. The physical and magnetic properties, MPI performance, and blood circulation time of these newly synthesized tracers were compared to those of two commercially available SPIONs that were not tailored for MPI but are used for MPI: ferucarbotran and PEG-coated Synomag^®^-D. The new tailored tracer has MPI sensitivity that is ~3-times better than the commercial tracer ferucarbotran and much longer circulation half-life than both commercial tracers (t_1/2_=6.99 h for the new tracer, vs t_1/2_=0.59 h for ferucarbotran, and t_1/2_=0.62 h for PEG-coated Synomag^®^-D).

## Introduction

Magnetic particle imaging (MPI) has attracted tremendous interest as a molecular imaging modality since it was first reported in 2005 1. In MPI, a uniform alternating magnetic field (AMF) is applied to a field of view while opposing magnets are used to create a quasistatic selection field gradient with a small field free region (FFR). Superparamagnetic iron oxide nanoparticles (SPIONs) located in the FFR respond to the applied AMF and generate a signal that can be recorded using pickup coils, while SPIONs outside the FFR are unable to respond to the AMF due to saturation caused by the selection field gradient 1, 2. The signal generated by SPIONs at the FFR is proportional to its mass and a quantitative 3D distribution of the SPIONs can be determined by moving the FFR to cover a field of view (FOV) of interest. Signal generation in MPI relies on the nonlinear superparamagnetic response of the SPIONs resulting in negligible signal from tissue, bones, and air gaps. Furthermore, there is negligible tissue attenuation of the magnetic fields used for MPI and of the signal generated by the SPIONs, resulting in images with negligible tissue depth limitations 1, 2. This combination of features makes MPI an ideal approach for unambiguous and sensitive non-invasive quantification of SPION biodistribution. In addition, because SPIONs can be used to label cells and other biomaterials, MPI has tremendous potential for applications such as cell tracking 3, nanoparticle drug delivery 4, image-guided procedures 5, monitoring tissue regeneration scaffolds 6, monitoring the brain 7, and blood pool imaging 8, 9.

The sensitivity and resolution achievable in MPI arise due to a combination of hardware, software, and the magnetic properties of the SPION tracer 10. The introduction of commercial pre-clinical MPI scanners has supported a wide range of studies seeking to apply MPI in novel biomedical settings and there is a tremendous need for SPION tracers with suitable MPI properties. Importantly, the physics of signal generation in MPI are distinct from that responsible for SPION contrast enhancement in magnetic resonance imaging (MRI). In MPI signal arises directly and solely from the non-linear magnetization response of SPIONs to the excitation field. In MRI, SPION contrast enhancement arises due to changes in proton relaxivity when they are in close proximity to SPIONs. Importantly, in MRI the SPIONs do not respond to the pulsed field because they are in a saturated state. As such, SPION tracers developed for MRI are not necessarily ideal for MPI. Furthermore, in addition to the magnetic properties of the SPIONs, surface modification and formulation must be tailored for specific applications. For example, there are several applications of MPI that would benefit from SPION tracers with long circulation lifetimes, including blood pool imaging 8, 9, functional MPI 11, cancer imaging 12, evaluate traumatic brain injury 7, and *in vivo* gut bleed detection 8. These considerations suggest a need for developing SPIONs with physicochemical and magnetic properties that are tailored for specific MPI applications.

Several commercially available SPIONs have been studied as MPI tracers. Ferucarbotran (an off-brand version of Resovist^®^) is a commercially available SPION contrast agent developed specifically for MRI that is commonly used for MPI studies 13, 14. However, it has been suggested that only 3% of the total iron mass from Resovist^®^ contributes to the MPI signal due to particle-particle interaction within carboxydextran coated core 1. Another commercially available tracer of potential use for MPI is Synomag^®^-D, which consists of multi-core SPIONs. Performance of Synomag^®^-D in MPI has been evaluated using a magnetic particle spectrometer (MPS) and the results suggested better performance compared to Resovist^®^ 15. Synomag^®^-D has been used to image flow in phantoms 16 and to label erythrocytes and cancer cells 17, 18. However, studies evaluating performance of Synomag^®^-D *in vivo* are lacking. SPION tracers have also been developed specifically for use in MPI. An example of a tracer developed specifically for MPI is LS-008, from LodeSpin Labs, LLC, which combined high sensitivity and resolution with a circulation half-life of ~105 min in mice 10, 19, 20. However, LS-008 is no longer available. Furthermore, while these tracers have been widely tested using academic prototype MPI scanners and with the Bruker pre-clinical MPI scanner, their performance has not been evaluated in the newer Magnetic Insight, Inc., MOMENTUM^TM^ pre-clinical scanner. Because SPION performance varies with the configuration of the magnetic field, the magnitude of the field gradient in the FFR, and the amplitude and frequency of the AMF used to excite the SPIONs, MPI performance of a given tracer is expected to vary from one type of scanner to another. The growing adoption of the MOMENTUM^TM^ MPI scanner suggests that comparative performance studies of MPI tracers using this scanner would be of value to the community.

Here we report synthesis, surface modification, and MPI performance of a new tracer (denoted as RL-1) tailored for MPI which possesses a long blood circulation half-life (~7 hour), suitable for blood pool imaging applications and other applications where long blood circulation time is desirable. The MPI performance and pharmacokinetics of the new RL-1 tracer are compared to those of the commercially available tracers ferucarbotran and Synomag^®^-D coated with polyethylene glycol. The SPIONs in this tracer were synthesized by thermal decomposition with addition of molecular oxygen 21, and subsequently coated with a covalently grafted layer of polyethylene glycol (PEG). Physical, magnetic, and hydrodynamic properties of the RL-1 tracer and the two commercial tracers were evaluated. The MPI performance (resolution, signal per unit Fe mass, and limit of detection) of all tracers and their pharmacokinetics in mice were evaluated using the MOMENTUM^TM^ MPI scanner.

## Methods

### Materials

Iron (III) acetylacetonate (>98% pure) and 3-aminopropyl triethoxysilane (APS, >98.0%) were purchased from TCI America (Portland, OR). Oleic acid (90% technical grade), docosane (90% pure), 1-octadecene (90% technical grade), polyethylene glycol monomethyl ether (mPEG, 5 kDa), sulfuric acid (99.999%), isopropyl alcohol (70%), tetra(ethylene glycol) dimethacrylate (TEGDMA, 90%), and 2,2′-Azobis(2-methylpropionitrile) (98%), potassium nitrate (>99%, ACS reagent), glycerol (>99%), were purchased from Sigma-Aldrich (St. Louis, MO). Toluene (>99.5%, ACS reagent), ethanol (200 proof), chromium trioxide (certified ACS), acetone (certified ACS), diethyl ether (certified ACS), hydrochloric acid (37% w/v), 1-ethyl-3-(3-dimethylaminopropyl) carbodiimide (EDC), activated charcoal (12-40 mesh), acetone (certified ACS), diethyl ether (ACS chemical, BHT stabilized), dichloromethane (99.6%, ACS reagent), nitric acid (Certified ACS Plus), potassium hydroxide (85%, ACS reagent), and CBQCA protein quantitation kit were purchased from Thermo Fisher Scientific (Waltham, MA). N-hydroxysulfosuccinimide (sulfo-NHS) was purchased from ProteoChem^TM^ (Hurricane, UT). Magnetic columns were purchased from Miltenyi Biotec (Germany). Ferucarbotran was purchased from Meito Sangyo Co., LTD (Japan). Synomag^®^-D, coated with PEG 25000-OMe, 50 nm, was purchased from micromod Partikeltechnologie GmbH (Germany). Copper TEM grid (carbon film only, 200 mesh) was purchased from TED PELLA, INC (Redding, CA).

### Particle synthesis

#### Synthesis of iron (III) oleate

A stoichiometrically defined iron oleate was prepared according to published work with modifications 21. Iron acetylacetonate (22.38 g, 63.36 mmol) and oleic acid (89.48 g, 316.80 mmol) were added to a 500 ml three-neck reactor. The flask was equipped with an overhead stirrer in the middle neck, a septum with a thermocouple and a stainless steel (SS) needle through the left neck. Argon (100 sccm) was supplied continuously through the SS needle during synthesis, using a mass flow controller. A condenser connecting to a chiller was attached in the right neck. A molten metal bath and temperature controller was used as the heating source. The molten metal was heated to 110 °C before pushing the reaction vessel into the molten metal bath. The reaction mixture was then heated to 310 °C under stirring at 350 rpm. Thirty minutes after the reaction mixture reached 300 °C, the reaction was stopped to obtain a dark brown waxy liquid. The resulting iron oleate was purged using argon and stored until use for nanoparticle synthesis.

#### Synthesis of magnetic iron oxide nanoparticles

Docosane (10.1 g, 32.23 mmol) and oleic acid (6.23 g, 22.06 mmol) were added to a 100 ml three-neck reactor. Separately, iron oleate was mixed with 1-octadecene (27.12 g, 107.40 mmol) to prepare a precursor with 0.22 M Fe. The flask was equipped with an overhead stirrer in the middle neck and a septum with a SS needle through the left neck. A molten metal bath and temperature controller was used as the heating source. The molten metal was heated to 110 °C before pushing the reaction vessel into the molten metal bath. The mixture was heated to 350 °C in 30 mins before the controlled addition of iron oleate precursor (1.98 to 2.64 mmol/h) using a syringe pump. Nitrogen (100 sccm) was supplied continuously through the SS needle, controlled using a mass flow controller until the reaction mixture reached 350 °C. Then, 140 sccm of 1% oxygen in argon mixture was introduced into the reactor headspace through the SS needle, controlled using a mass flow controller. Uniform mixing at 350 rpm was maintained throughout the reaction. The precursor drip was stopped after 4 to 5 hours and the reactor was removed from the molten metal bath. Toluene and ethanol in a 2:3 volume ratio were used to precipitate nanoparticles from the crude synthesis product. Purified oleic acid coated particles were suspended in toluene and stored at 4 °C.

### Particle coating

#### PEG-silane synthesis

A polyethylene glycol-silane conjugate (PEG-silane) was synthesized via a two-step procedure. First, mPEG (5 kDa) was converted to mPEG acetic acid (mPEG-COOH) using a strong oxidizing agent 22. Briefly, 50 g of mPEG was dissolved in 400 mL of acetone. Jones reagent (prepared using 70 g of chromium trioxide in 500 mL of deionized water and 71 mL of sulfuric acid) was used to oxidize mPEG. Once the mPEG was dissolved in acetone, 16.1 mL of Jones reagent was added and allowed to react for 24 hours. Approximately, 5 mL of isopropyl alcohol was added to stop the reaction and 5 g of activated charcoal was added to remove impurities. The chromium salts and activated charcoal were removed using vacuum filtration. The acetone solution containing the oxidized mPEG was concentrated using a rotary evaporator. The concentrated mixture of mPEG-COOH was re-dissolved in 50 mL of 1 M HCl. The polymer was then extracted to the organic phase by liquid-liquid extraction using 150 mL dichloromethane. The extraction allows for removal of chromium trioxide since it is insoluble in dichloromethane. The solution was concentrated by rotary evaporation. The concentrated mPEG-COOH was precipitated using cold diethyl ether. The mPEG-COOH was then dried in a vacuum oven at room temperature. Proton nuclear magnetic resonance (NMR) spectroscopy was used to ascertain there was full conversion of mPEG to mPEG-COOH. Second, mPEG-COOH was amidated by reaction with APS to obtain PEG-silane. Briefly, mPEG-COOH was weighed and melted in an oil bath set to 60 ºC. Then, APS was added to the melted PEG at a 1:1 molar ratio. The mixture was allowed to react for 2 hours at 120 °C and 500 mbar. The PEGsilane was then cooled to room temperature and collected. The resulting PEG-silane was analyzed through gel permeation chromatography (GPC).

#### Ligand exchange

SPIONs were coated with PEG-silane using ligand exchange, replacing the oleic acid on the surface of the SPIONs with PEG-silane, following procedures similar to Zhu et al. 23. Briefly, 0.7 g of PEG-silane was dissolved in 4 mL of dry toluene. Once the PEG-silane was dissolved, 2 mL of SPIONs at 2.5 mg Fe3O4 per mL and 28 µL of APS were added and mixed. The solution was capped and allowed to react overnight, approximately 16 hours, in a heating block set at 100 °C. The next day, the PEG-silane coated SPIONs were precipitated out of solution using cold diethyl ether. The sample was centrifuged and supernatant discarded. The SPIONs were resuspended in acetone and precipitated again with cold diethyl ether twice. The precipitate was then dried in a vacuum oven at room temperature overnight. The following day, PEG-silane coated SPIONs were resuspended in water and dialyzed to remove excess PEG-silane. For further purification, particles were purified using magnetic columns. The resulting nanoparticles were backfilled with additional PEG-COOH using EDC-NHS chemistry 24. The number of remaining primary amines on the particles was quantified using the CBQCA protein quantification kit, following the manufacturer's protocol. Once the number of amines were determined, a ratio of 1:2 amine to carboxylic acid was used. The mPEG-COOH was suspended in water and pH adjusted to 5.0. EDC was added at a 1:2 carboxylic acid:EDC ratio and allowed to react for 15 minutes. Then, sulfo-NHS was added at a 1:1 ratio of EDC to sulfo-NHS. The pH of the solution was slowly adjusted to 7.0 and reacted for 15 minutes. Last, the nanoparticle solution was added and the pH adjusted to 9.0. The mixture reacted overnight and was purified using a magnetic column. Finally, the nanoparticles were sterilized using a 0.22 µm PES syringe filter.

### Physical and magnetic characterization

#### Transmission Electron Microscopy

Images of iron oxide particles sampled on 200-mesh copper grids with carbon film were acquired using a FEI Talos F200i S/TEM. Physical diameters (D_p_) were obtained by analyzing the images using Fiji 25. Reported size distribution statistics and histograms are based on at least 2000 particles for RL-1 nanoparticles, or at least 400 particles for ferucarbotran and Synomag^®^-D.

The number median diameter (D_pg_) and geometric standard deviation (ln σ_g_) of the particle size distribution were obtained by fitting the size distribution histograms to the lognormal distribution (n_N_(D_p_)) using 21:


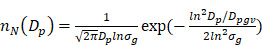
(1)

D_pg_ was converted to a volume median diameter (D_pgv_) using 21:


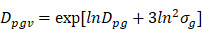
(2)

The arithmetic volume weighted mean diameter (D_pv_) and standard deviation (σ) were calculated using 21:


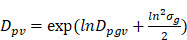
(3)


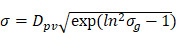
(4)

#### Dynamic Light Scattering and Zeta Potential

A Brookhaven Instruments 90Plus/BI-MAS dynamic light scattering and zeta potential measurement instrument, operating at a scattering angle of 90° at room temperature, was used to determine the hydrodynamic size and zeta potential of the SPIONs. For hydrodynamic diameter measurements, particles were suspended at 1 mg/mL in deionized water. The zeta potential of the particles was measured in a 1 mM KNO_3_ solution at pH 7, adjusted with nitric acid and potassium hydroxide.

#### Magnetometry

Magnetic characterization was performed with the particles suspended in water at 300 K to obtain magnetization data for the purpose of bimodal magnetic diameter fitting. Particle concentration was ~1 mg Fe/ml, according to the 1,10-phenanthroline spectrophotometric assay 26.

Nanoparticles were also embedded in a TEGDMA matrix using a technique described previously in order to perform more detailed magnetic characterization 27. These characterizations included magnetization versus magnetic field (MH) curves taken at 295, 305 and 315 K, plotted as a function of the ratio of magnetic field to absolute temperature, to verify superparamagnetic behavior. Additionally, the zero field cooled/field cooled measurements were used to obtain blocking temperatures for the estimation of magnetic anisotropy constant. The MH curves obtained at 315 K were used to obtain the monomodal magnetic diameter estimate used in determination of the anisotropy constant as well. To prepare samples embedded in TEGDMA, a concentrated nanoparticle suspension in water was mixed with TEGDMA monomer at a particle concentration of 0.1 wt %. Then, the initiator 2,2′-Azobis(2-methylpropionitrile) was added at a concentration of 0.05 wt %, and crosslinking was performed by heating the mixture at 70 °C for 6 h.

DC equilibrium magnetization curves of the particles in water and in the hard polymer matrices were obtained using a magnetic property measurement system (MPMS-3) superconducting quantum interference device (SQUID) magnetometer (Quantum Design, Inc. CA, USA). Samples were mounted in the instrument using PTFE sample holders for suspensions and plastic straws for polymer matrices.

#### Magnetic Diameter fitting for liquid samples

The volume-weighted median magnetic diameters (D_mv_) and geometric deviation (ln σ_g_) of the iron oxide nanoparticles suspended in water at 300 K were obtained by fitting the measured magnetization data M(H) to the Langevin function L(α) for superparamagnetism, weighted using a bimodal lognormal size distribution. The single population lognormal weighting for the Langevin function suggested by Chantrell et al. 28, was modified to a bimodal distribution by considering that the particle magnetic diameter population was well-represented by the sum of two single modal lognormal distributions. In the equations below, M_S_ is the saturation magnetization of the sample, ϕ_1_is the mass fraction of the first diameter distribution, n_v1_(D_m_) and n_v2_(D_m_) are the lognormal distribution functions, α is the Langevin parameter, μ_0_is the permeability of free space, M_d_ is the domain magnetization (446,000 A/m for bulk magnetite 29), k_B_ is Boltzmann's constant, and T is the measurement temperature. D_mv,1_ and D_mv,2_ are the volume weighted median diameters of the two magnetic diameter distributions, and ln σ_g,1_ and ln σ_g,2_ are the geometric deviations. The fit was performed in MATLAB^®^ (MathWorks, MA, USA) using a non-linear regression model. The arithmetic volume weighted mean diameter (D_mv_) and standard deviation (σ) were calculated using Equation (3) and (4).


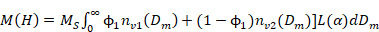
(5)


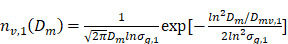
(6)


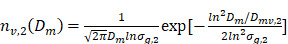
(7)


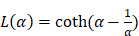
(8)


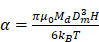
(9)

#### Estimation of effective anisotropy constant

The volume-weighted median magnetic diameters (D_mv_) and geometric deviation (ln σ_g_) of the iron oxide nanoparticles embedded in solid matrix were obtained by fitting the magnetization data to the Langevin function L(α) for superparamagnetism, weighted using a monomodal lognormal size distribution, as suggested by Chantrell et al. 28. Note that bimodal size distributions were used in the case of liquid samples because they match the measured magnetization data with more fidelity. In contrast, here we chose a monomodal distribution because the model used to estimate the effective anisotropy constant does not account for multiple size distributions. In equations (10) and (11) below, n_v_(D_m_) is the lognormal distribution function, M_S_ is the saturation magnetization of the sample, and α is the Langevin parameter, defined in equation (9).


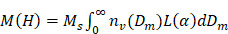
(10)


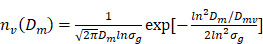
(11)

The fit was performed in MATLAB using a non-linear regression model. Zero field cooled and field cooled (ZFC-FC) magnetization measurements were made to obtain the blocking temperature (T_B_) for each nanoparticle. Samples embedded in polymer matrices were prepared as described above. At the start of the measurements, samples were first heated to 400 K at zero field, and then cooled to 4 K at zero field. A field of 10 Oe was applied and the magnetization measured as the temperature was swept at 2 K/min from 4 K to 400 K in the ZFC portion of the curve. Then, for the FC portion of the curve, the sample was cooled to 4 K at 2 K/min while the magnetization of the sample was measured. The value of the blocking temperature T_B_ was estimated by applying a simple parabolic fit to the portion of the ZFC curve where the peak in measured magnetization occurred. Equation (12) below was then used to calculate the effective anisotropy constant using the Néel model, accounting for the dispersity in magnetic diameters 27. Here, K_m_ is the effective magnetic anisotropy constant of the particles, k_B_ is the Boltzmann's constant, T_B_ is the blocking temperature, D_mv_ is the volume weighted median magnetic diameter from the monomodal fit performed above, τ_obs_ is the observation time, τ_0_ is the attempt frequency (assumed widely to be 10^-9^ s), ln σ_g_ is the geometric deviation of the magnetic diameter distribution obtained above, and T_rate_ is the temperature sweep rate of our ZFC/FC measurements, equal to 2 K/min in all measurements performed.


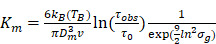
(12)

#### Dynamic Magnetic Susceptibility

The dynamic magnetic susceptibility of all tracers in liquids (200 μl of total volume) of different viscosities were measured using a DynoMag AC susceptometer (Rise Research Institutes, Sweden) in a small amplitude oscillating magnetic field at a constant temperature and as a function of the frequency of the oscillating magnetic field. Measurements were made in deionized water and in a 65% w/w glycerol in water solution (viscosity = 0.0125 Pa s) in order to evaluate the mechanism of magnetic relaxation of the particles.

### MPI performance

#### Sample holder design

To image each sample in the MPI scanner, novel sample holders were designed using the online, three-dimensional (3D) computer aided design program Onshape (Onshape, MA, USA) and 3D printed with the Form 3 stereolithography printer (Formlabs, MA, USA). Each sample holder was designed as a removable part that fits inside a customized 3D printed bed which was attached to the MPI scanner arm. This custom bed design is shown in Figure S1. The parts used in MPI scans were printed using Clear V4 resin (Formlabs, MA, USA) with layer sizes ranging from 25-100 µm.

The design of the capillary tube holder used for limit of detection (LoD) testing is shown in Figure S2. This design holds up to 7 tubes oriented vertically and arranged in a linear pattern along the z-scan direction in the FOV. The tube bores are 2.82 mm in diameter with a center-to-center spacing of 10.35 mm.

Relax scan measurements performed on samples in 0.2 mL microcentrifuge tubes utilized sample holders which held the tubes vertically. Similar to the capillary tube holders, these microcentrifuge tube holders each hold up to 7 tubes centered within the 80 mm sample holder length. Each microcentrifuge tube rests in a 6.82 mm diameter bore, and the bores have a center-to-center spacing of 11.20 mm. The vertical configuration of this model is shown in Figure S3.

#### MPI measurements

To determine the LoD for each SPION, we first determined the iron concentration using the 1,10-phenanthroline colorimetric assay 26. Dilution series were prepared using a dilution factor of two, with concentrations ranging from 1000 µg_Fe_/ml to 15 µg_Fe_/ml. All samples consisted of 1 µL of solution (containing 1 µg_Fe_ to 15 ng_Fe_) in a capillary tube (1/32” ID) placed parallel to the y-axis in the field of view (FOV). Each concentration was acquired in triplicate by placing three capillary tubes featuring the same iron mass in the FOV (6 × 12 cm). MPI scans were acquired with the MOMENTUM^TM^ scanner (Magnetic Insight, CA, USA) using high-sensitivity (3 T/m) multichannel scan mode (x- and z-channel scans).

Images were analyzed using MATLAB^®^ (MathWorks, MA, USA) in-house algorithms in which the region of interest (ROI) was selected and maximum signal intensity was obtained. Images were also analyzed using 3D Slicer 30, 31. The LoD was evaluated several ways. First, an LoD was calculated by analyzing the background signal from empty scans, the limit of blank (LoB), and the maximum intensity signal of samples at low concentrations, using the equations 32:


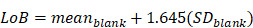
(13)


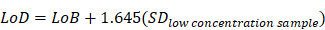
(14)

The LoD was also evaluated based on calculation of mean signal to noise ratio (mSNR), calculated as the ratio of the mean signal intensity in the ROI to the standard deviation of the background region for each scan. Finally, the LoD was evaluated by inspection of the individual scans to confirm that the signal corresponded to the dilution samples and not to background signal fluctuations.

For relax scan measurements, 10 µL of each particle (RL-1, Ferucarbotran, and Synomag^®^-D) were placed in a 0.2 mL microcentrifuge tube, and the sample was centered in the FOV. Then, the x-space point spread function (PSF) was measured using the RELAX^TM^ modality in the MOMENTUM^TM^ scanner. The PSF was normalized by the iron mass to facilitate comparison of different particles. The signal intensity was reported by normalizing the system reported amplitude using iron mass and the FWHM is the system reported value.

### Tracer blood circulation time evaluated via MPI

#### Animal models

All animal procedures were conducted according to the protocols approved by the Institutional Animal Care and Use Committee at the University of Florida. Female Balb/c (6 weeks old) were obtained from Envigo (Indianapolis, IN). All animals were acclimatized for at least one week prior to experimentation.

#### Tracer administration and imaging

Female Balb/c mice 8-11 weeks old were used for all the experiments. SPIONs were dispersed in sterile PBS 1x and filtered with 0.22 µm PES filters prior to intravenous administration. Mice were injected with 200 µL of 1 mg_Fe_/mL as a bolus injection in the lateral tail vein using a 28 G insulin syringe (*n*=3). Mice were anesthetized immediately with 4-5% isoflurane in an induction chamber and then maintained at 1-2% for the duration of the imaging period. Imaging was performed using MOMENTUM^TM^ scanner (Magnetic Insight, CA, USA) for quantitative imaging. Animals were placed in a 3D printed animal bed (Figure S4) designed to be compatible with the MOMENTUM^TM^ MPI scanner and the Perkin Elmer IVIS SpectrumCT imager used to acquire anatomical CT images. Imaging was done in high sensitivity/high resolution (5.7 T/m) scan mode and a 12 cm × 6 cm × 6 cm FOV. Scans were performed at the following time intervals for RL1-C (0, 0.5, 1, 2, 4, 6, 12, 24, and 48 h), ferucarbotran (every 2 min for the first 1 h and at 24 h), and Synomag^®^-D (0, 0.5, 1, 2, 4, 6, and 24 h). Acquisition times were 3 min for 2D scans and 42 minutes for 3D scans.

#### Quantitative analysis of MPI

SPIONs distribute rapidly in the circulatory system once injected. Two regions of interest (ROI) were selected: one for the heart to represent particles in circulation and another for the liver, as the compartment in which most SPIONs will deposit. For each image, a threshold value was used to eliminate inherent background signal from the bed and instrument. The threshold value was determined using the maximum intensity pixel of an empty bed scan. After this threshold was applied to all images, an ROI was drawn over the heart and liver/spleen region. The size of the ROIs was equal for all the images. The reported MPI signal was the total signal in the ROI. To determine tracer half-life, MPI signal for each ROI was fitted to a simple one compartment model using a nonlinear least-squares method.


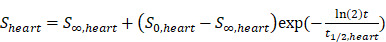
(15)


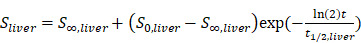
(16)

where S_heart_ is the signal in the heart ROI, *S_liver_* is the signal in the liver ROI, *t* is time, *S_∞,heart_* and *S_∞,liver_* are the long-time signals in the heart and liver ROI, accounting for residual background signal, S_0,heart_ and *S_0,liver_* are the initial signals in the heart and liver ROI, and *t_1/2,heart_* and *t_1/2,liver_* are the characteristic half-lives for particle clearance from the blood circulation and for particle accumulation in the liver, respectively. The 95% confidence interval was evaluated for the estimated half-lives. All image processing and analysis was performed using MATLAB^®^ (MathWorks, MA, USA).

#### Image registration

Anatomic CT (IVIS SpectrumCT, Perkin Elmer, MA, USA) reference images were acquired on anesthetized animals in standard-one mouse mode with voxel size of 150 µm and resolution of 425 µm (20 ms exposure time, 440 AI X-Ray filter). Image registration was established using fiducials that contained a mixture of SPIONs (MPI tracer) and Omnipaque^TM^ (CT tracer) as markers to align the MPI data with the CT maximum projection image. MPI-CT 2D images were registered using MATLAB^®^ (MathWorks, MA, USA) while 3D registration and visualization was performed using 3D Slicer the landmark registration module and using maximum intensity projection for volume rendering 30, 31.

## Results

### Comparison of tracer physical and magnetic properties

Iron oxide nanoparticles were synthesized through the thermal decomposition of iron oleate in the presence of molecular oxygen. Three batches of particles (RL-1A, RL-1B, RL-1C) were obtained to illustrate reproducibility over physical and magnetic properties. Then their properties were compared to two commercial tracers, ferucarbotran and Synomag^®^-D coated with PEG.

**Figure 1:**
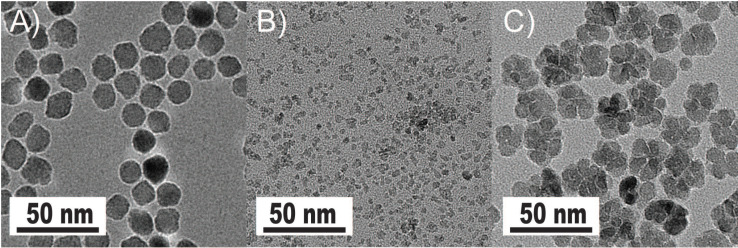
** Tracer evaluation via transmission electron microscopy.** A) RL-1C. B) Ferucarbotran. C) Synomag®-D.

The synthesized and commercially obtained SPIONs were investigated using bright field transmission electron microscopy (TEM). Because PEGsilane (RL-1 particles), carboxydextran (ferucarbotran) and PEG-OMe modified dextran (Synomag^®^-D) coatings give negligible contrast under electron microscopy, the physical morphology and size distribution are representative of the iron oxide crystal cores of each tracer. As shown in Figure 1, the RL-1C particles (A), representative of the RL-1 particles, are single core with narrow size distribution, while ferucarbotran particles (B) consists of small agglomerated cores and Synomag^®^-D particles (C) consist of heterogeneous nanocrystal clusters. Volume weighted mean physical diameters and standard deviation of all tracers are summarized in Table 1. RL-1 particles had core diameters and standard deviation of 22.6 ± 2.0 nm (RL-1A), 20.7 ± 2.7 nm (RL-1B), and 21.4 ± 2.4 nm (RL-1C). Ferucarbotran had core diameters and standard deviation 9.6 ± 2.9 nm, and Synomag^®^-D particles had core diameters and standard deviation of 28.6 ± 9.4 nm.

**Figure 2:**
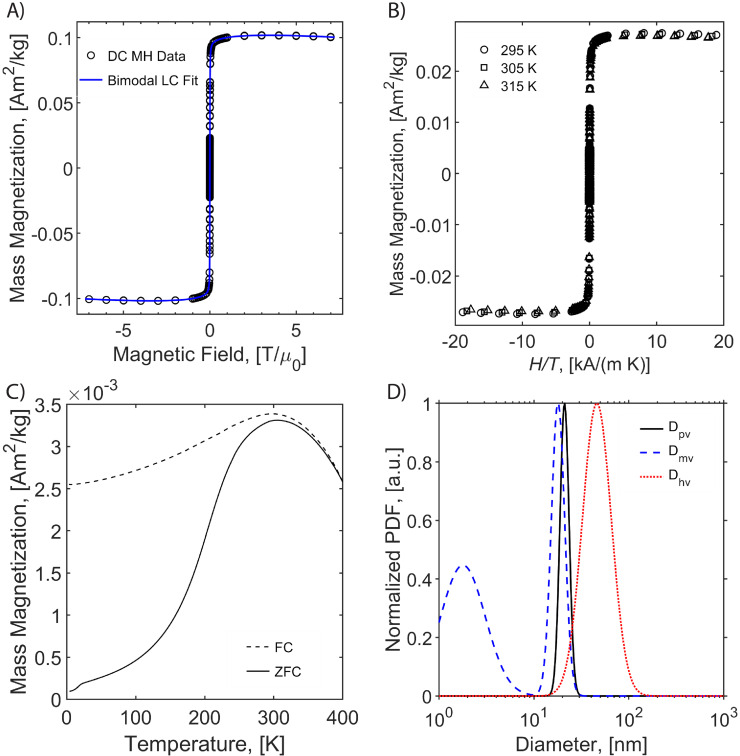
** Magnetic characterization of RL-1C.** A) MH curve at 300 K PEG coated RL-1 particles in water. B) MH curves at 295, 305, 315K in TEGMA. C) ZFC/FC at 10 Oe in TEGMA. D) Physical, hydrodynamic and magnetic diameter distribution.

Magnetic properties of the particles are critical for their performance in MPI. Therefore, the magnetic properties of all tracers were evaluated using SQUID magnetometry. As seen in the magnetization versus magnetic field (MH) curves, superparamagnetism is apparent for all tracers either in liquid (Figure 2A, Figure S5A, and Figure S6A) or in solid matrix (Figure 1B, Figure S5B, and Figure S6B), as there is a very large initial susceptibility, negligible coercivity or remanent magnetization, and magnetic saturation is reached at relatively small magnetic fields. Superposition of the MH measurements for three different temperatures in solid TEGMA further suggests superparamagnetic behavior (Figure 1B, Figure S5B, and Figure S6B). The magnetization data was fitted to the Langevin function weighted using a bimodal lognormal size distribution. The results suggest that all three tracers behave as if possessing two populations of particles with different magnetic size distributions. The arithmetic volume-weighted mean magnetic diameter, standard deviation, and volume fraction of each population are summarized in Table 1. Magnetic diameter distributions are also illustrated in Figure 2D (RL-1C) and Figure S7 (all tracers studied). The RL-1 particles had consistent magnetic distributions with the majority (ϕ_1_ ~ 0.9) of the particles having magnetic diameters of 18.1 ± 4.2 nm, 17.1 ± 2.1 nm, and 18.4 ± 3.0 nm. The remaining ϕ ~ 0.1 of the particles had a relatively small magnetic diameter of 2.5 ± 1.6 nm, 3.1 ± 1.4 nm, and 2.8 ± 1.6 nm. The larger magnetic diameter population is consistent with the physical size distribution of the RL-1 particles. For ferucarbotran, ϕ_1_ ~ 0.81 of the magnetic volume had a size of 7.6 ± 4.1 nm, while the remaining ϕ ~ 0.19 had a magnetic diameter of 22.1 ± 4.4 nm, consistent with other reports in the literature 33. The small magnetic diameter fraction in ferucarbotran is consistent with the physical size, although the magnetic diameter distribution is broader. It has been suggested that the population with larger magnetic diameter arises due to magnetic interactions between individual crystals in the dextran matrix 33. For Synomag^®^-D, ϕ_1_ ~ 0.84 of the magnetic volume had a magnetic diameter of 19.3 ± 3.7 nm and ϕ ~ 0.16 had a magnetic diameter of 8.2 ± 3.0 nm. These numbers are similar (if slightly smaller) to the physical size of the particle clusters and the small cores that make up each cluster. Interestingly, the larger magnetic size has a narrower distribution relative to the physical size distribution of the clusters that make up Synomag^®^-D.

The temperature dependence of magnetization was evaluated using zero field cooled/field cooled (ZFC-FC) measurements by measuring the magnetization of a sample embedded in solid matrix as a function of temperature. These measurements yield the blocking temperature of the nanoparticles, indicative of the temperature above which the majority of the particles become superparamagnetic. The resulting ZFC-FC curves are shown in Figure 2C (RL-1C), Figure S5C (ferucarbotran), and Figure S6C (Synomag^®^-D). The blocking temperatures were found to be 307 K, 226 K, and 310 K, for RL-1C, ferucarbotran, and Synomag^®^-D, respectively. The blocking temperatures were then used to calculate the effective anisotropy constant of all tracers. The anisotropy constant contributes to the particle relaxation mechanism and ultimately affects MPI performance 34. It was found that the effective anisotropy constant of RL-1 (30 kJ/m^3^) is similar to that of Synomag^®^-D (30 kJ/m^3^) and both are larger than ferucarbotran (21 kJ/m^3^). All three of these values are higher than the K_m_ value for bulk magnetite reported in literature (13.5 kJ/m^3^) 35. We note that recent work suggests that true blocking temperatures for particle systems with size and shape polydispersity are significantly lower than the peak of the ZFC curve 36.

The arithmetic volume weighted mean hydrodynamic diameter and standard deviation values of all three nanoparticles were evaluated using dynamic light scattering (DLS) and are summarized in Table 1. RL-1C particles had a hydrodynamic diameter of 55 ± 20 nm, ferucarbotran had a hydrodynamic diameter of 65 ± 28 nm, and Synomag^®^-D had a hydrodynamic diameter of 60 ± 18 nm. Physical, magnetic, and hydrodynamic size distributions are illustrated in Figure 2D for RL-1C and for all particles in Figure S7. The zeta potential was determined at physiological pH of 7.2-7.4. RL-1C particles had a zeta potential of ζ = -7.6 mV, ferucarbotran had a zeta potential of ζ = -12.9 mV, and Synomag^®^-D particles had a zeta potential of ζ = -6.5 mV.

Dynamic magnetic susceptibility measurements were made for all tracers to study their relaxation mechanism (Figure S8). Magnetic nanoparticles respond to time-varying magnetic fields by internal dipole rotation (i.e., Néel relaxation) or physical particle rotation (i.e., Brownian relaxation), with each mechanism being sensitive to the properties of the particles and the surrounding medium. It is important to characterize the mechanism of SPION magnetic relaxation because this can impact their MPI performance 37. Because the viscosity of the carrier liquid will affect the relaxation time of particles undergoing Brownian relaxation but not of particles undergoing Néel relaxation, we measured dynamic magnetic susceptibility spectra in water (η = 0.001 Pa s) and in water-glycerol solutions (η = 0.0152 Pa s) 27. For particles undergoing Brownian relaxation, one would expect a significant (one-decade) shift toward lower frequency in the DMS spectra of particles in the water-glycerol solution, compared to particles in water. No such shift is observed in the DMS spectra for all tracers, suggesting they all undergo Néel relaxation (Figure S8). Furthermore, peaks were not observed at the calculated Brownian peak frequencies (dotted lines in Figure S8), further suggesting that the particles undergo primarily Néel relaxation 27.

### Comparison of tracer MPI performance

**Figure 3:**
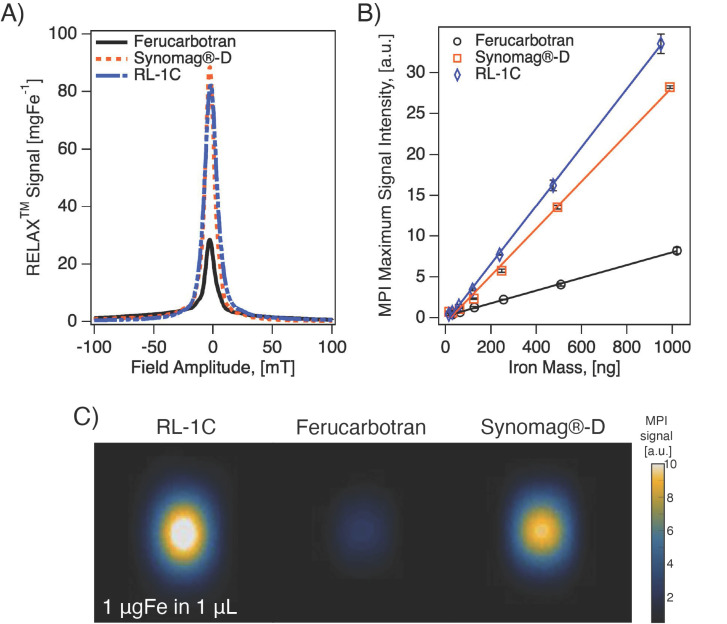
** MPI properties of commercially available tracers and RL SPIONs.** A) PSF obtained using relax module in MOMENTUM^TM^ scanner shows signal intensity of SPIONs. B) Serial dilution shows linear relationship of iron mass and MPI signal for all three tracers in 2D high-sensitivity scan modes. C) 2D MPI maximum intensity projection for 1 mgFe in 1 µL of solution for all three tracers.

We evaluated the MPI performance of in-house synthesized RL-1 tracers and compared these with commercial ferucarbotran and Synomag^®^-D. MPI performance was characterized according to the point-spread function (PSF) using the MPI RELAX^TM^ module and by evaluating the limit of detection for a dilution series of samples imaged in 2D high sensitivity multichannel scan mode (Figure 3). The RELAX^TM^ module in the MOMENTUM^TM^ measures the magnetization for a field sweep between -100 mT and 100 mT using a 16 mT, 45 kHz excitation field. The PSF is related to the derivative of the Langevin function and characterizes the performance of SPIONs in x-space MPI 37, 38. The PSF was used to determine the signal intensity (peak height) per iron mass, which is a measure of expected particle sensitivity in MPI, and to obtain the full-width half-maximum (FWHM), which relates to the expected resolution in MPI. Representative PSF results for all three tracers are shown in Figure 3A. Table 1 summarizes the PSF properties for all particles used in this study. Figure S9 shows PSF for three independent batches of RL-1 particles, showing reproducibility in MPI performance. The MPI maximum signal intensity values for the three batches of RL-1 particles range from 70 to 83 mg_Fe_^-1^, while the corresponding values are 26 mg_Fe_^-1^ for ferucarbotran and 88 mg_Fe_^-1^ for Synomag^®^-D. In terms of resolution, the three batches of RL-1 particles had FWHM ranging from 11.4 mT to 13.0 mT, while the corresponding values were 11.2 mT for ferucarbotran and 9.2 mT for Synomag^®^-D. The spatial FWHM in units of mm were calculated assuming a field gradient of 5.7 T/m, resulting in expected spatial resolutions of 2.0 to 2.3 mm for RL-1 particles, 2.0 mm for ferucarbotran, and 1.6 mm for Synomag^®^-D. Using the PSF we observed that RL-1 particles and Synomag^®^-D have similar sensitivity in the MPI. In terms of resolution, RL-1 and ferucarbotran are similar while Synomag^®^-D is slightly better.

In contrast to expectations based on the PSF peak intensities, Figure 3B suggests higher signal per unit mass for RL-1C particles imaged in 2D high-sensitivity scan mode (3.055 T/m gradient strength, 15.5 mT RF excitation in the x-channel and 20.5 mT RF excitation in the z-channel). However, although Figure 3B suggests a difference in signal per unit mass, the limit of detection (LoD) calculated using equations (13) and (14) is similar for RL-1C and Synomag^®^-D nanoparticles. The calculated LoD was 28.31 ng_Fe_ for RL-1C particles, 29.81 ng_Fe_ for Synomag^®^-D, and 57.75 ng_Fe_ for ferucarbotran. The sensitivity for each tracer was further evaluated by inspection of the z-channel images obtained in the high sensitivity scans for each dilution for each tracer. Figure S10 shows these scans. The corresponding mean signal to noise ratio (mSNR) for each tracer and each dilution are shown in Figure S11. According to this analysis, the RL-1 sample can still be detected (mSNR=4.5) at 30 ng_Fe_, while for Synomag®-D at 31 ng_Fe_ we obtained mSNR = 1.7 and for Ferucarbotran at 32 ng_Fe_ we obtained mSNR = 1.8. For the next larger tracer mass in the series, we obtained mSNR = 3.6 for Synomag®-D at 62 ng_Fe_ and mSNR = 3.6 for Ferucarbotran at 64 ng_Fe_. We note that while the two methods of LoD determination agree for RL-1, additional fiducials of intermediate masses would be necessary for Synomag®-D and ferucarbotran.

### Tracer pharmacokinetics in mice

The pharmacokinetics of the three tracers was evaluated in mice and blood circulation and liver accumulation half-lives were estimated using single-compartment models. Particles were administered via tail vein injection. *In vivo* MPI 2D images of each tracer in mice (n=3) were taken at specified time intervals. These images were analyzed by image registration of MPI and CT images and positioning ROIs around the heart and around the liver/spleen (denoted as liver). The MPI total intensity was obtained from the ROIs for each animal for all tracers at all time points. Representative images for one mouse from the RL-1C group for all time points are shown in Figure S12.

**Figure 4:**
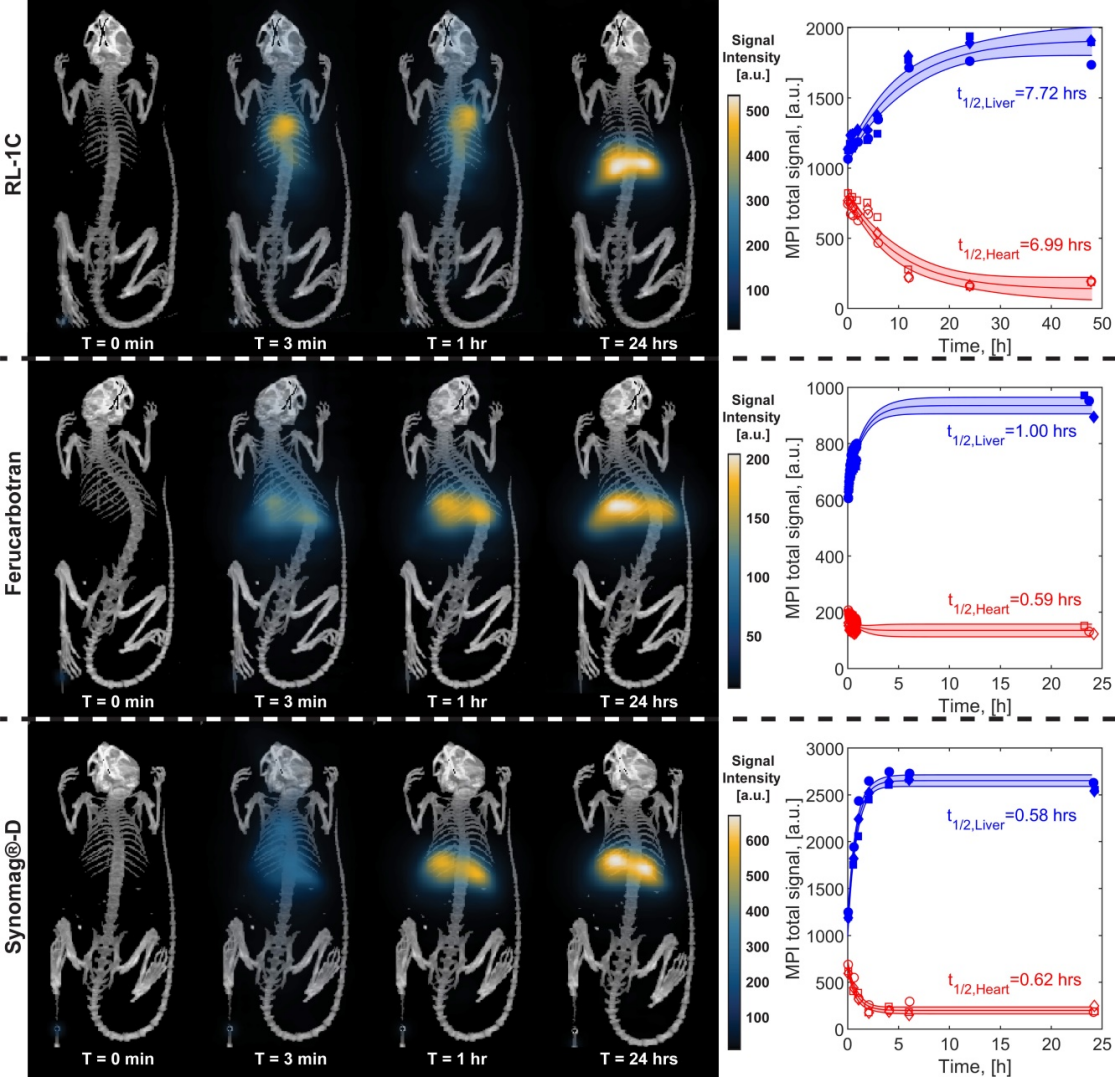
** Representative MPI/CT images at short and long time points and MPI signal intensity in heart and liver ROIs as a function of time for each tracer.** Each animal is shown with different markers. Data was fitted to a nonlinear least square fit single compartment model to estimate blood circulation half-life.

Figure 4 shows representative MPI scans, registered to CT for anatomical reference, at similar time points and the associated MPI total intensity data for all three animals at all time points for each tracer. Immediately after intravenous injection of the tracer, one should observe whole-body distribution of the tracer, with greater signal intensity in organs with larger blood volume, such as the heart and lungs. Over time, the vascular signal will decrease, while the signal in the liver and spleen will increase. The images of all mice right before injection (t=0 min) show no signal, as expected since there is no tracer. Three minutes after injection (t=3 min), RL-1C tracers were seen to be distributed throughout the body, with the greatest signal corresponding to the heart. Similar distribution was observed for Synomag^®^-D, albeit with a weaker heart signal and more significant liver signal compared to RL-1C. In contrast, ferucarbotran was observed primarily in the liver and spleen, even at the relatively short time point of t = 3 min. One hour after injection, the signal from RL-1C was still primarily in the heart and lungs, whereas almost all the signal for ferucarbotran and Synomag^®^-D came from the liver and spleen. By 24 hours, signal for all tracers was consistent with accumulation in the liver and spleen. Three-dimensional visualization of the tracers using MPI registered with CT for anatomical reference can be found in Movie S1 for RL-1C at 1 h, Movie S2 for RL-1C at 24 h, Movie S3 for ferucarbotran at 24 h, and Movie S4 for Synomag^®^-D at 24 h. Three-dimensional MPI images for the two commercial tracers were not acquired at 1 h due to their faster clearance rate relative to the time it takes to obtain a 3D image. Qualitatively, these results suggest that RL-1 particles have longer blood circulation than the commercially available particles.

Blood circulation half-life for all three tracers was calculated from fitting the MPI total signal intensity for the heart and liver ROI to a simple first-order one compartment model. Figure 4 shows the data for each tracer, for all time points collected for each mouse. The shaded region corresponds to the 95% confidence interval for the fit prediction and indicates good fitting for all the tracers. RL-1C showed the longest circulation half-life of t_1/2,heart_ = 6.99 h, while ferucarbotran had a t_1/2,heart_ = 0.59 h and Synomag^®^-D had a t_1/2,heart_ = 0.62 h. The same algorithm used to calculate blood circulation half-life was used to assess the rate of accumulation of tracers in the liver and spleen. The results were t_1/2,liver_ = 7.72 h for RL-1C, t_1/2,liver_ = 1.00 h for ferucarbotran, and t_1/2,liver_ = 0.58 h for Synomag^®^-D. Both qualitatively and quantitatively, RL-1 tracers had a much longer blood circulation half-life than the commercial tracers ferucarbotran and Synomag®-D coated with PEG.

## Discussion

The results of this study show how thermal decomposition of iron-oleate precursor in the presence of molecular oxygen can be leveraged to obtain single-core SPIONs with reproducible enhanced MPI performance. The three tracers studied had similar hydrodynamic diameters according to dynamic light scattering, but consisted of SPIONs with different crystal sizes and morphology. Interestingly, although RL-1 consisted of ~20-22 nm single-core crystals and Synomag®-D consisted of ~29 nm multicore crystals, they had similar magnetic diameter distributions, blocking temperatures, and estimated effective anisotropy constants. We believe this is the reason why they had similar MPI sensitivity performance, albeit with better expected resolution for Synomag®-D. This is consistent with the notion that MPI performance is determined principally by the magnetic properties of the tracer. Of relevance, both RL-1 and Synomag®-D had the majority (80-90%) of their magnetic volume assigned to a population with a magnetic diameter of ~18-19 nm, with the rest of the magnetic volume corresponding to a population with a magnetic diameter of ~2-4 nm. In contrast, only 20% of the magnetic volume in ferucarbotran corresponded to a population with a magnetic diameter of ~22 nm, with the rest of the magnetic volume corresponding to a population with a magnetic diameter of ~4.4 nm. We believe this is the reason why RL-1 and Synomag®-D have ~3X better MPI signal per Fe mass than ferucarbotran.

Ideal tracers for blood pool imaging using MPI should yield sufficient vascular signal after a single tracer injection for a period long enough to allow diagnosis. Long circulating nanoparticles can lead to better planning and diagnostics for different applications such as cancer detection and imaging, blood pool evaluation, monitoring bleeding, and for functional MPI in the brain. Blood circulation half-life was determined for RL-1 and the two commercially available tracers (ferucarbotran and Synomag^®^-D) in female Balb/c mice. The pharmacokinetic data was fitted to a standard first-order one compartment model. The assumptions of the one compartment model are that the tracer distributes and equilibrates rapidly throughout the vascular system and that elimination begins immediately after administration. The results shown in Figure 4 appear consistent with a one compartment model. Importantly, RL-1C showed the longest blood circulation half-life of ~7 hours.

Ferucarbotran has been studied widely as a contrast agent for MRI. In another MPI study performed in rats using ferucarbotran, no signal was observed in the heart or jugular veins 10 minutes after injection, suggesting that the blood circulation half-life of ferucarbotran in rats is much shorter than 10 minutes 20. The blood circulation half-life of ferucarbotran has also been evaluated using magnetic particle spectroscopy of blood samples in mice and rabbits, and the half-life was determined to be 5-10 minutes 39, 40. However, that study was limited due to the small amount of blood that could be obtained and the sensitivity of the equipment used to detect tracer signal. In a human study of ferucarbotran as an MRI contrast agent, the tracer exhibited a biexponential blood concentration decay, with a half-life of 3.9-5.8 min for the fast initial phase accounting for roughly 80% of the injected dose and a second half-life of 2.4-3.6 h for the second phase 41. In another study using the Bruker pre-clinical MPI scanner, which is able to perform scans at 21.5 milliseconds per frame, the authors observed biexponential blood concentration decay for ferucarbotran in FVB mice with first fast clearance phase of 0.63 minute and a slower phase of 13 minutes 42. We did not observe biexponential decay in blood tracer concentration. However, by our first imaging time point, 3 minutes after injection, most of the ferucarbotran seemed to have accumulated in the liver already. As such, our measurements most likely missed an initial, fast signal decay. This would suggest that our analysis of the data obtained is representative of the second phase of ferucarbotran clearance from blood, which would be in agreement with the study by Kaul et al.^42^, which suggests that the second decay phase starts within 3 minutes. Furthermore, it is relevant to point out that the studies by Hamm *et al.* 41 were in humans, whereas that of Kaul et al. and our study were in mice. It has been shown that nanoparticle tracers have longer blood circulation times in humans than in mice, as it takes about one minute for the tracer to pass the whole circulatory system in a human, while in mice it takes about 5-10 seconds 43.

Use of Synomag®-D in MPI is growing, as they provide better MPI performance than ferucarbotran in terms of sensitivity and resolution. However, the blood circulation time of Synomag^®^-D particles has not been reported. Our results suggest that Synomag^®^-D has a blood circulation half-life of 37 minutes, compared to 31 minutes for ferucarbotran.

Dynamic light scattering and zeta potential measurements suggests that all three tracers have similar hydrodynamic size distributions (~65 nm for ferucarbotran, ~60 nm for Synomag®-D, and ~55-75 nm for RL-1) and negative charge (-12.9 mV for ferucarbotran, -6.5 mV for Synomag®-D, and -7.6 mV for RL-1). However, the three tracers differ significantly in terms of the nature of their surface coating. Depending on their coating, nanoparticles can be recognized by the mononuclear phagocytic system and taken up and removed from circulation. Ferucarbotran is coated with carboxymethyl dextran, which is a complex carbohydrate that can easily be phagocytosed by macrophages due to its overall negative charge and due to the action of mannose/lectin receptors, which recognizes the nanoparticle and initiate endocytosis. Coating nanoparticles with PEG is widely adopted to prolong their blood circulation time. Although Synomag®-D is advertised as coated with PEG (25 kDa) for prolonged circulation time in blood, our results suggest a relatively short blood circulation half-life of ~0.62 hours. We attribute this to the fact that, based on information provided by the supplier, Synomag^®^-D is actually initially coated with a dextran shell before conjugating PEG onto the dextran. Depending on the extent of coating, the dextran layer on Synomag®-D may be recognized by macrophages and taken up just like in ferucarbotran. In contrast, the RL-1 nanoparticles are coated with a covalently bonded brush of PEG, which we believe is responsible for their comparatively longer blood circulation time.

A possible limitation of the present study is the reliance on MPI signal in regions of interest *in vivo* to estimate SPION blood circulation and liver/spleen accumulation dynamics, without comparison to *ex vivo* quantification by other means, such as inductively coupled plasma mass spectroscopy. However, correlation between *in vivo* and *ex vivo* MPI SPION quantification and linearity of MPI signal with SPION concentration have been demonstrated previously 12, 20. While comparison to other means of quantification of iron, such as inductively coupled plasma mass spectrometry, would be desirable, this would require animal euthanasia at each time point of interest, significantly increasing the burden of research animal use. In fact, this is precisely one of the reasons why MPI is so attractive for quantitative tracking of SPIONs *in vivo*.

## Conclusions

Magnetic particle imaging, an emerging molecular imaging modality, has great potential in applications such as blood pool imaging, functional brain imaging, cancer imaging, evaluating traumatic brain injury, and *in vivo* gut bleed detection. Long circulating nanoparticles can lead to better planning and diagnostics for these applications. In this study, MPI-tailored SPION tracers were synthesized through thermal decomposition with molecular oxygen, followed by coating with covalently bonded PEG. Physical and magnetic properties of the synthesized tracers were evaluated and compared to commercial tracers (ferucarbotran and Synomag^®^-D). The synthesized tracer RL-1 had similar MPI performance compared to Synomag^®^-D, which was attributed to their similar magnetic diameter distributions and blocking temperatures. Both tracers were ~3-times better in MPI signal per tracer mass than ferucarbotran result. The blood circulation half-life of the RL-1 tracer was also evaluated and compared to the two commercially available tracers. Analysis of in-vivo MPI in mice study suggests that RL-1 has a blood circulation half-life of 6.99 h, much longer than that of ferucarbotran (0.59 h) and Synomag^®^-D (0.62 h). These results suggest that RL-1 tracers are excellent candidates for MPI applications that require long blood circulation.

## Supplementary Material

Supplementary figures.

Supplementary moie/video S1.

Supplementary moie/video S2.

Supplementary moie/video S3.

Supplementary moie/video S4.

## Figures and Tables

**Table 1:** Comparison of physical and magnetic properties and MPI performance of commercial tracers and RL-1 tracers tailored for MPI

	Ferucarbotran	Synomag^®^-D	RL-1A	RL-1B	RL-1C
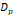 , [nm]	9.6	28.6	22.6	20.7	21.4
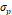 , [nm]	2.9	9.4	2.0	2.7	2.4
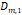 ^a^, [nm]	7.6	8.2	2.5	3.1	2.8
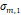 ^a^, [nm]	4.1	3.0	1.6	1.4	1.6
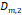 ^a^, [nm]	22.1	19.3	18.1	17.1	18.4
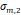 ^a^, [nm]	4.4	3.7	4.2	2.1	3.0
ø_1_	0.8	0.2	0.1	0.1	0.1
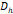 , [nm]	65	60	54	76	55
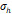 , [nm]	28	18	25	35	20
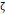 , [mV]	-12.9	-6.5	-	-	-7.6
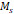 , [Am^2^/kg]	32	56	-	-	44
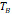 , [K]	226	320	-	-	307
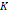 , [kJ/m^3^]	21	30	-	-	30
RELAX^TM^ Signal, [mg Fe^-1^]	25.8	87.8	77.3	69.5	82.6
RELAX^TM^ FWHM, [mT]	11.2	9.2	11.4	13.0	11.9
FWHM^b^, [mm]	1.96	1.61	2.00	2.28	2.09

a: Magnetic diameter distribution parameters were obtained in DI water suspension. b: FWHM [mm] is calculated using the gradient value of 5.7 T/m.

## Abbreviations

SPION: superparamagnetic iron oxide nanoparticle
PEG: polyethyelene glycol
MPI: magnetic particle imaging
AMF: alternating magnetic field
FFR: field free region
FOV: field of view
MRI: magnetic resonance imaging
MPS: magnetic particle spectrometer
APS: 3-aminopropyl triethoxysilane
m-PEG: polyethylene glycol monomethyl ether
TEGDMA: tetra(ethylene glycol) dimethacrylate
EDC: 1-ethyl-3-(3-dimethylaminopropyl) carbodiimide
sulfo-NHS: N-hydroxysulfosuccinimide
SS: stainless steel
PEGsilane: polyethylene glycol-silane conjugate
mPEG-COOH: mPEG acetic acid
NMR: nuclear magnetic resonance
GPC: gel permeation chromatography
MH: magnetization versus magnetic field
ZFC-FC: zero field cooled and field cooled
3D: three-dimensional
LoD: limit of detection
ROI: region of interest
LoB: limit of blank
PSF: point spread function
TEM: transmission electron microscopy
DLS: dynamic light scattering
FWHM: full-width half-maximum
mSNR: mean signal to noise ratio
