# Supplementary material for: Long circulating tracer tailored for magnetic particle imaging
Source: Nanotheranostics. 2021 Mar 24;5(3):348–61. doi: 10.7150/ntno.58548 (PMC8040827; doi:10.7150/ntno.58548)
Supplement: Supplementary file 1 — Supplementary figures. [file ntnov05p0348s1.pdf]

Title: Long circulating tracer tailored for magnetic particle imaging

Authors:

Sitong Liu[1,*], Andreina Chiu-Lam[1,*], Angelie Rivera-Rodriguez[2,*], Ryan DeGroff[2], Shehaab Savliwala[1], Nicole Sarna[2], Carlos Rinaldi[1,2,+]

Affiliations:

1. Department of Chemical Engineering, University of Florida, Gainesville, FL 32611
2. J. Crayton Pruitt Family Department of Biomedical Engineering, University of Florida, Gainesville, FL 32611-6131

*These authors contributed equally to this work.

[+]Corresponding author

Supplementary information

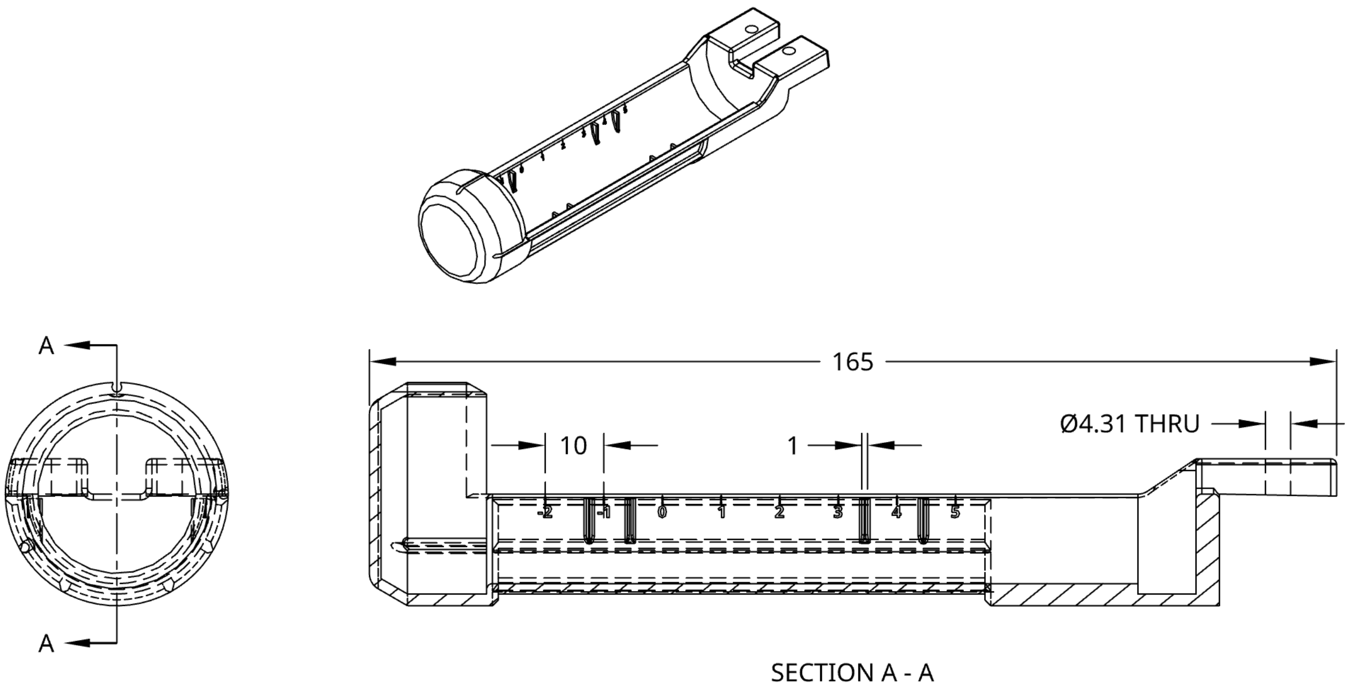

Figure S1. Custom 3D-printed sample bed for phantom and SPION sample MPI studies. Dimensions of this custom-designed sample bed were derived from Magnetic Insight's 45mL sample tube design and modified to fit the custom sample holders that were designed and 3D-printed for this study.

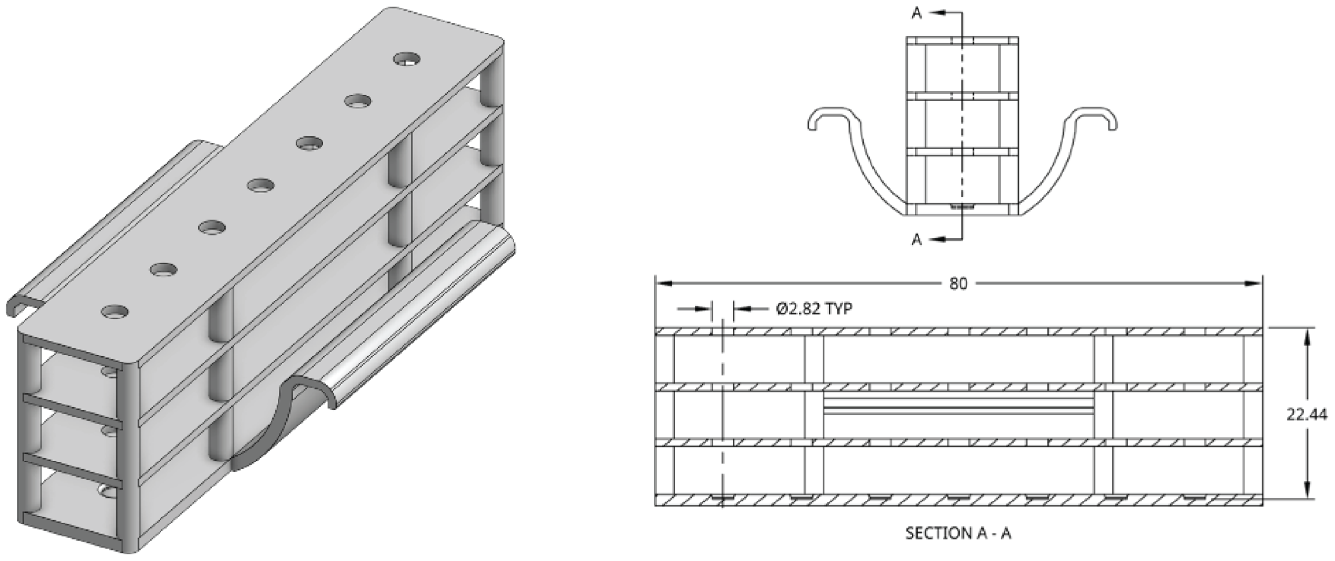

Figure S2. Vertical capillary tube holder designed and 3D-printed for LoD studies. Custom sample holders were designed in Onshape and 3D printed using Formlabs Clear V4 resin with the Form 3 SLA 3D printer.

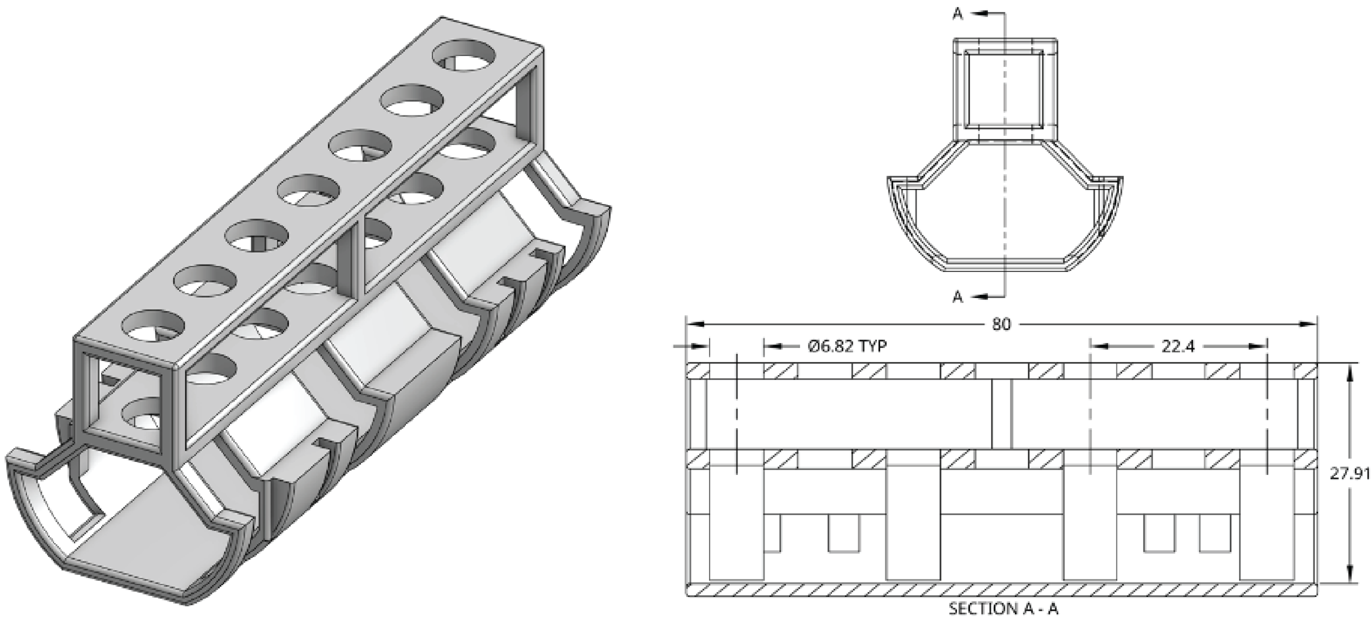

Figure S3. Vertical 0.2 mL microcentrifuge tube holder designed and 3D-printed for MPI relax scan measurements. The design accommodates 7 total tubes, with the entire lengths of 4 tubes able to be seen from a side view through exposed spaces. These 4 exposed spaces are 27.91 mm in vertical height with a center spacing of 22.4 mm.

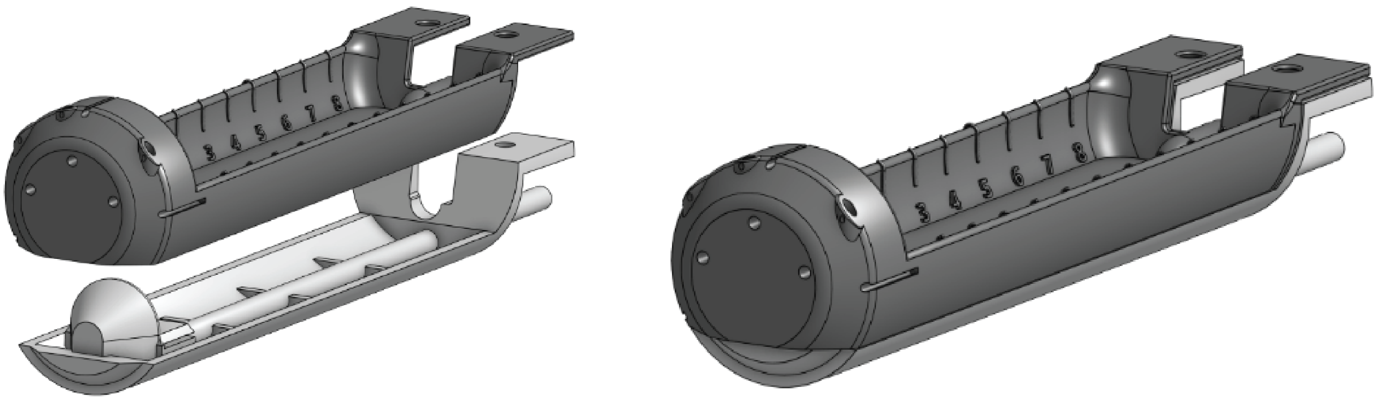

Figure S4. Rendering of two-part animal bed assembly designed and 3D-printed for MPI animal studies. The top and bottom animal bed parts (left) assemble a whole animal bed (right) that can be installed on Magnetic Insight's MPI scanner. The top part was designed with a flat bottom for ease of transfer of the mice from MPI to CT. The bottom part houses an integrated tubing system for isoflurane administration during imaging.

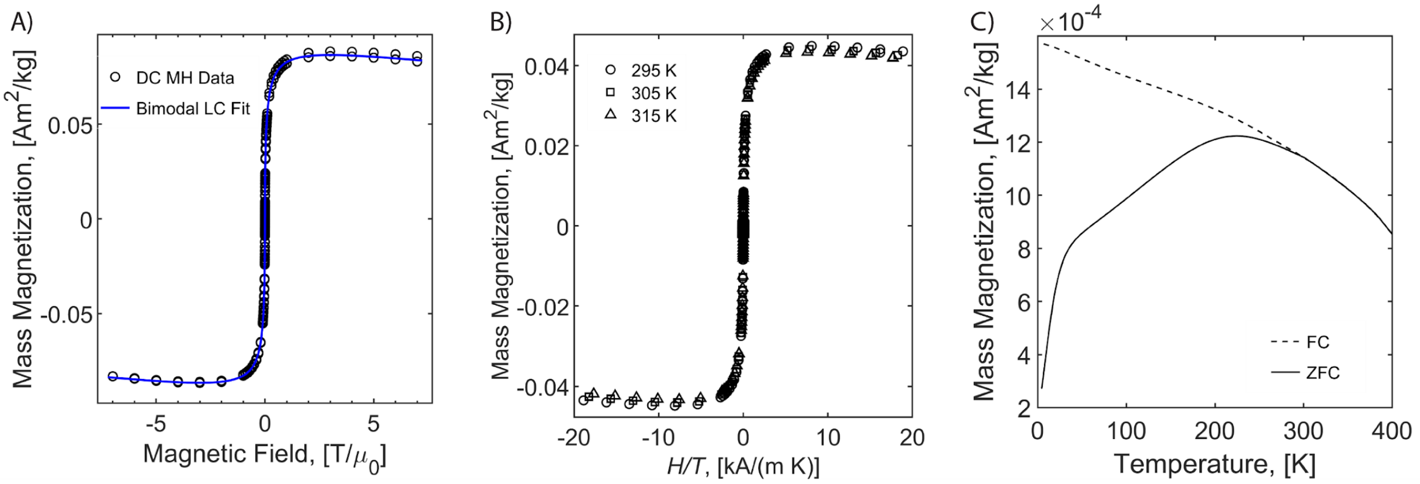

Figure S5. Magnetic characterization of ferucarbotran. A) MH curve at 300 K for ferucarbotran in water. B) MH curves at 295, 305, 315K for ferucarbotran in TEGMA C) ZFC/FC at 10 Oe for ferucarbotran in TEGMA.

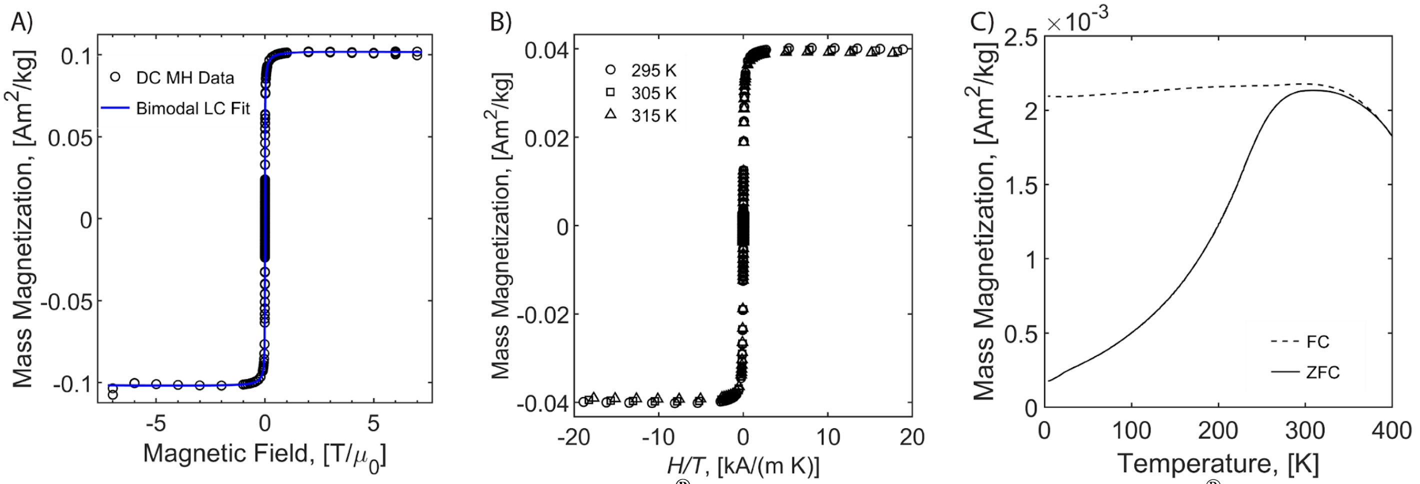

Figure S6. Magnetic characterization of Synomag®-D. A) MH curve at 300 K for Synomag®-D in water. B) MH curves at 295, 305, 315K for Synomag®-D in TEGMA C) ZFC/FC at 10 Oe for Synomag®-D in TEGMA.

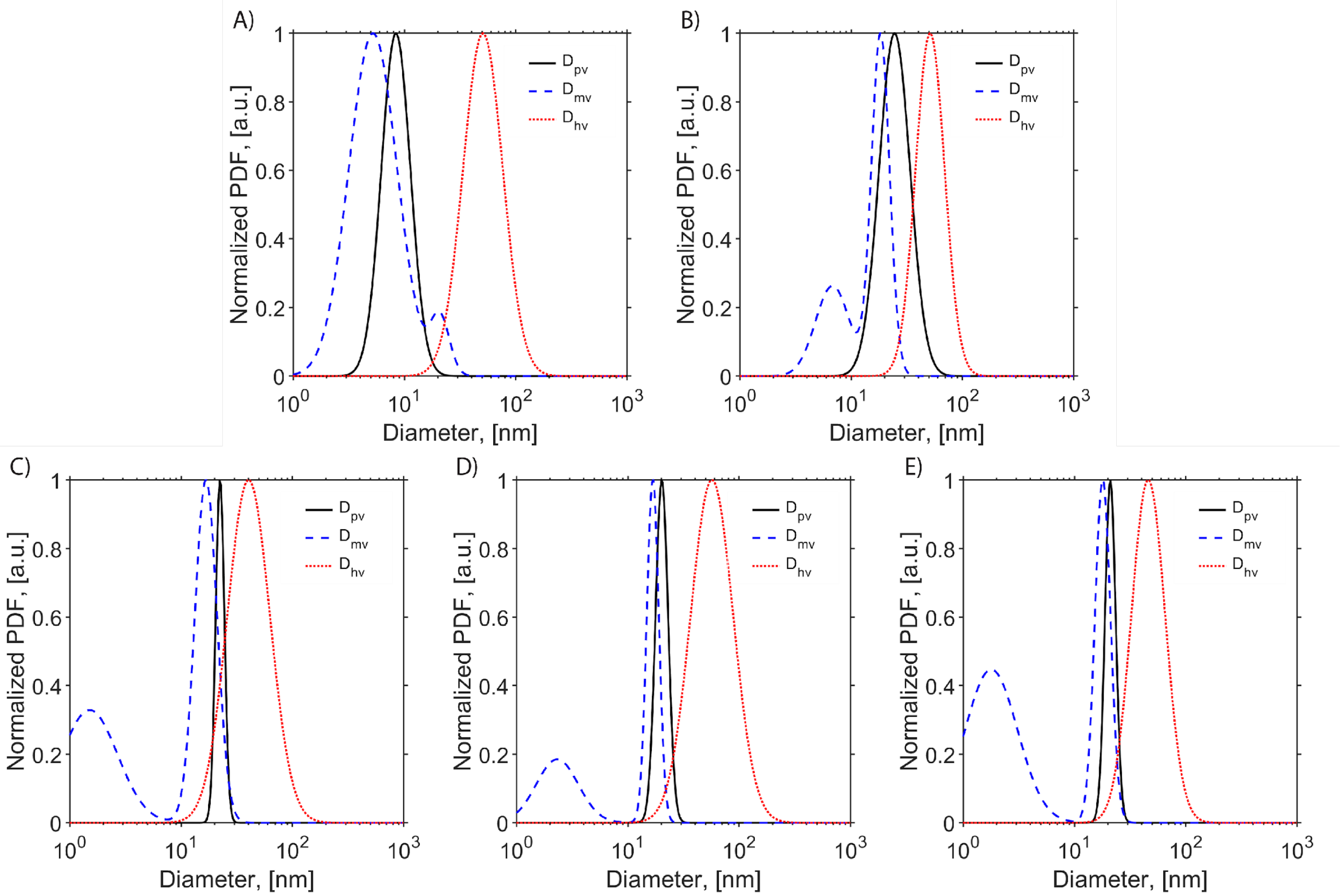

Figure S7. Physical, magnetic, and hydrodynamic size distribution histograms for A) ferucarbotran. B) Synomag®-D. C) RL-1A. D) RL-1B. E) RL-1C.

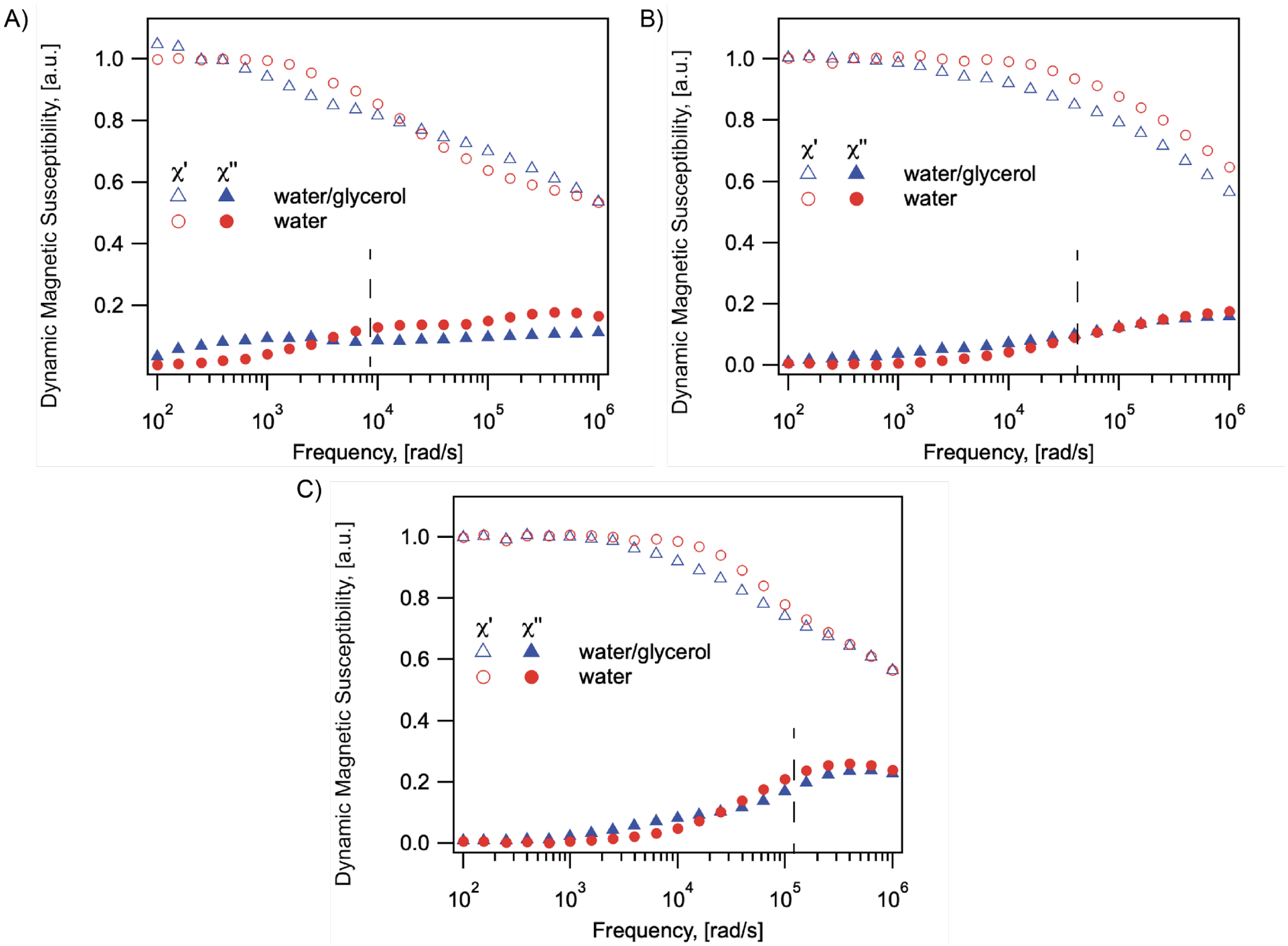

Figure S8. Dynamic magnetic susceptibility characterization of relaxation mechanism for all three tracers. A) ferucarbotran. B) Synomag®-D. C) RL-1C. in water (red circle) or water and glycerol mixture (blue triangle, 65 wt% of glycerol). Dash line indicating peak frequency position for each particle assuming Brownian Relaxation dominant, based on their hydrodynamic diameter.

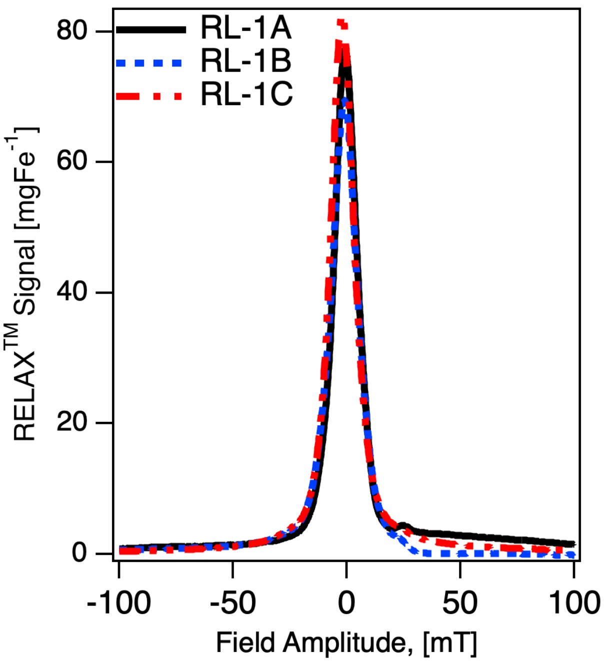

Figure S9. PSF for several RL-1 tracer batches demonstrate reproducibility in MPI performance.

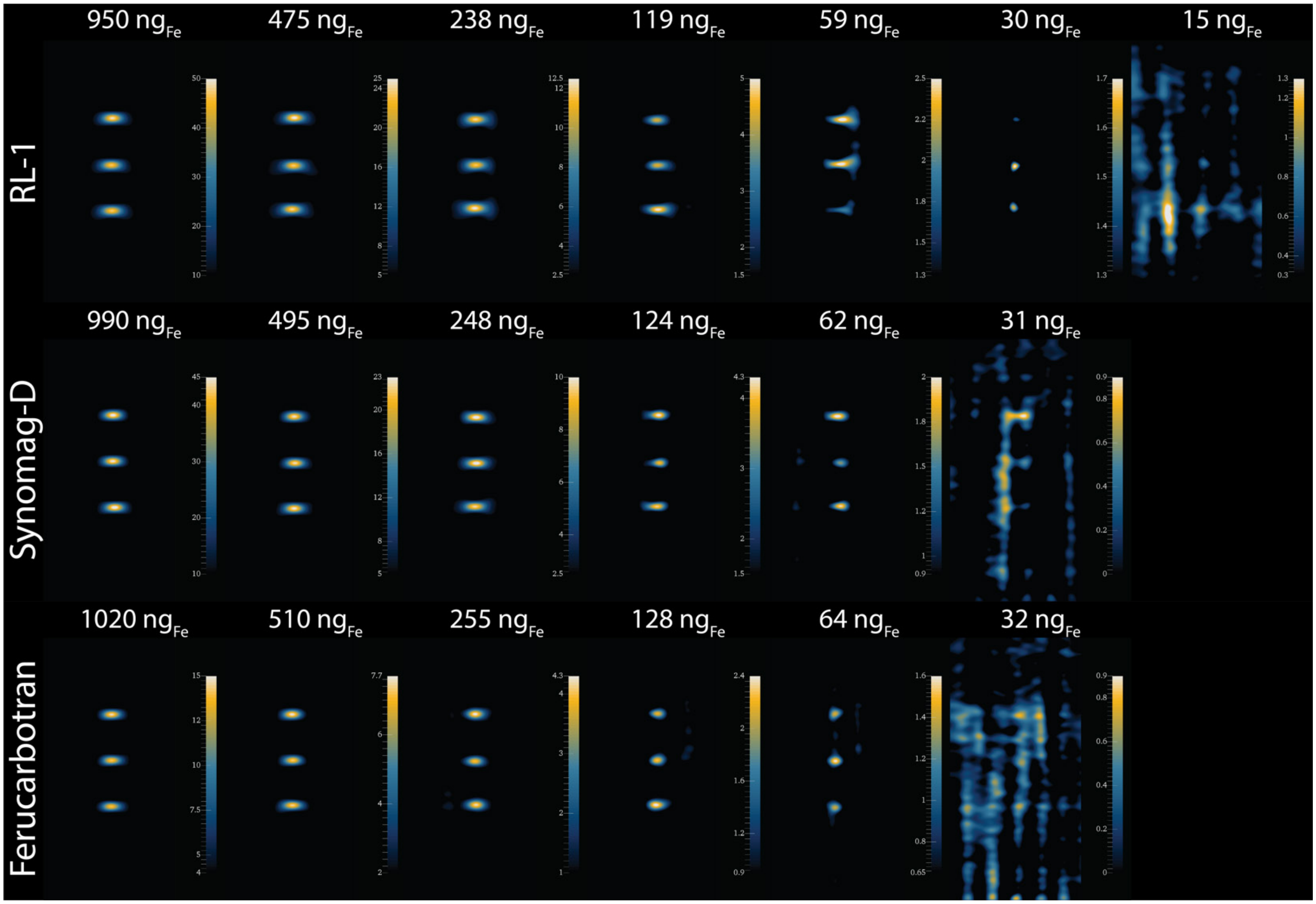

Figure S10. Representative 2D MPI z-channel dilution series for commercially available tracers and RL-1C. MPI scans were acquired in high-sensitivity.

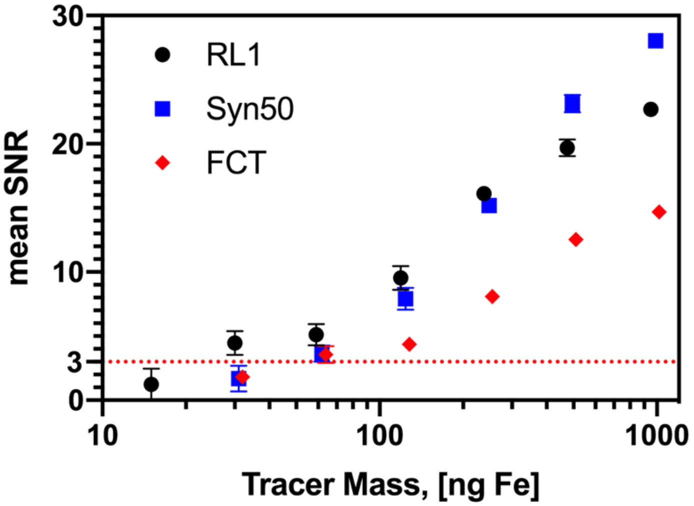

Figure S11. Mean signal to noise ratio (mSNR) as a function of tracer mass.

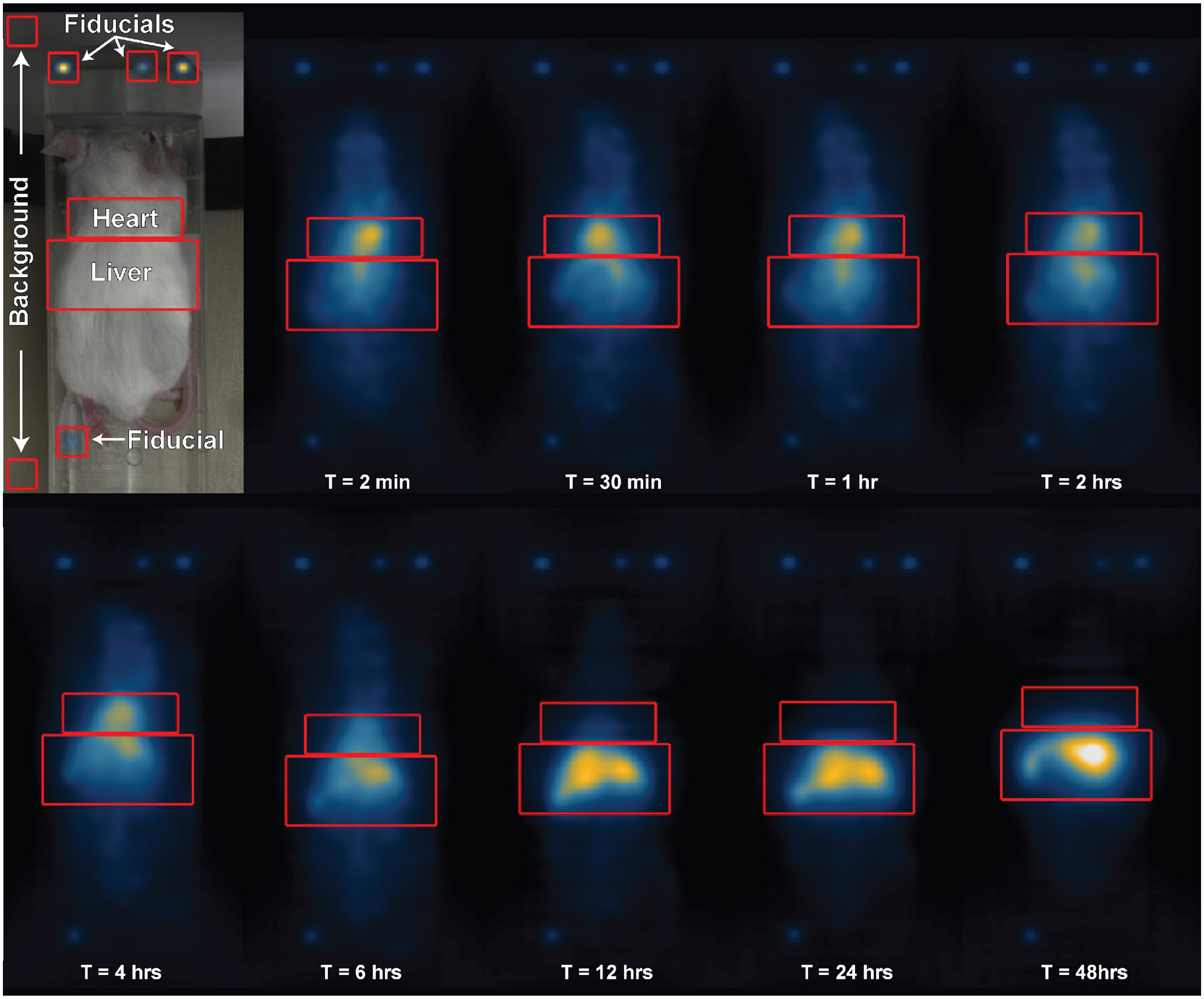

Figure S12. Collage showing representative MPI images to each time point for one mouse for RL-1C tracer. The coregistered optical image with MPI is for reference to the signals observed in the MPI scans at different times. The ROIs for the heart and liver/spleen were all the same size across all times.

Movie S1. Video of mouse injected with RL-1C tracer 1 hour after administration. The video corresponds to coregistered CT image with 3D MPI scan.

Movie S2. Video of mouse injected with RL-1C tracer 24 hours after administration. The video corresponds to coregistered CT image with 3D MPI scan.

Movie S3. Video of mouse injected with ferucarbotran tracer 24 hours after administration. The video corresponds to coregistered CT image with 3D MPI scan.

Movie S4. Video of mouse injected with Synomag®-D tracer 24 hours after administration. The video corresponds to coregistered CT image with 3D MPI scan.
