# Supplementary figures and images for: Long circulating tracer tailored for magnetic particle imaging
Source: Nanotheranostics. 2021 Mar 24;5(3):348–61. doi: 10.7150/ntno.58548 (PMC8040827; doi:10.7150/ntno.58548)

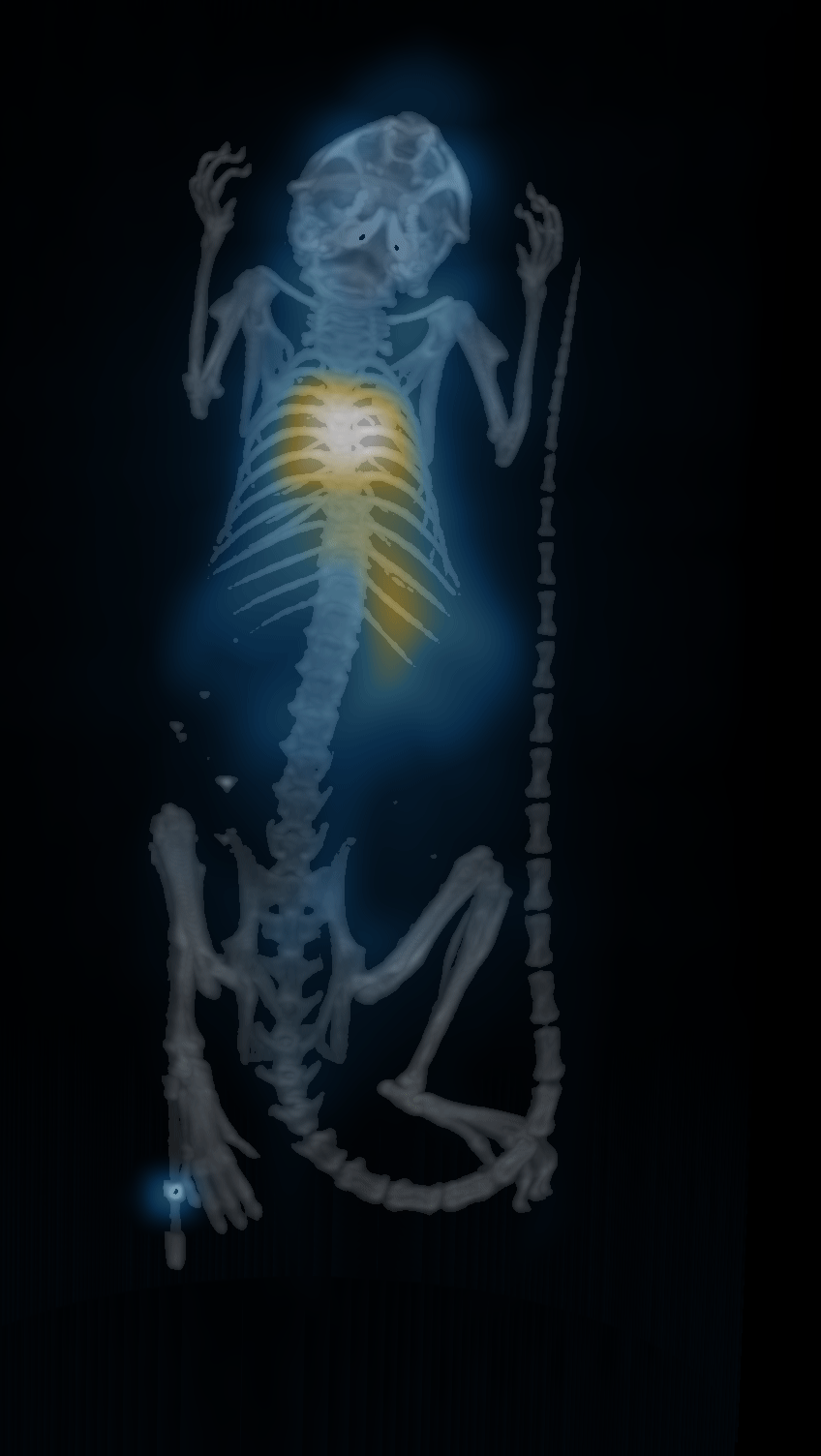

Supplement: Supplementary file 2 — Supplementary moie/video S1. [file ntnov05p0348s2.gif]

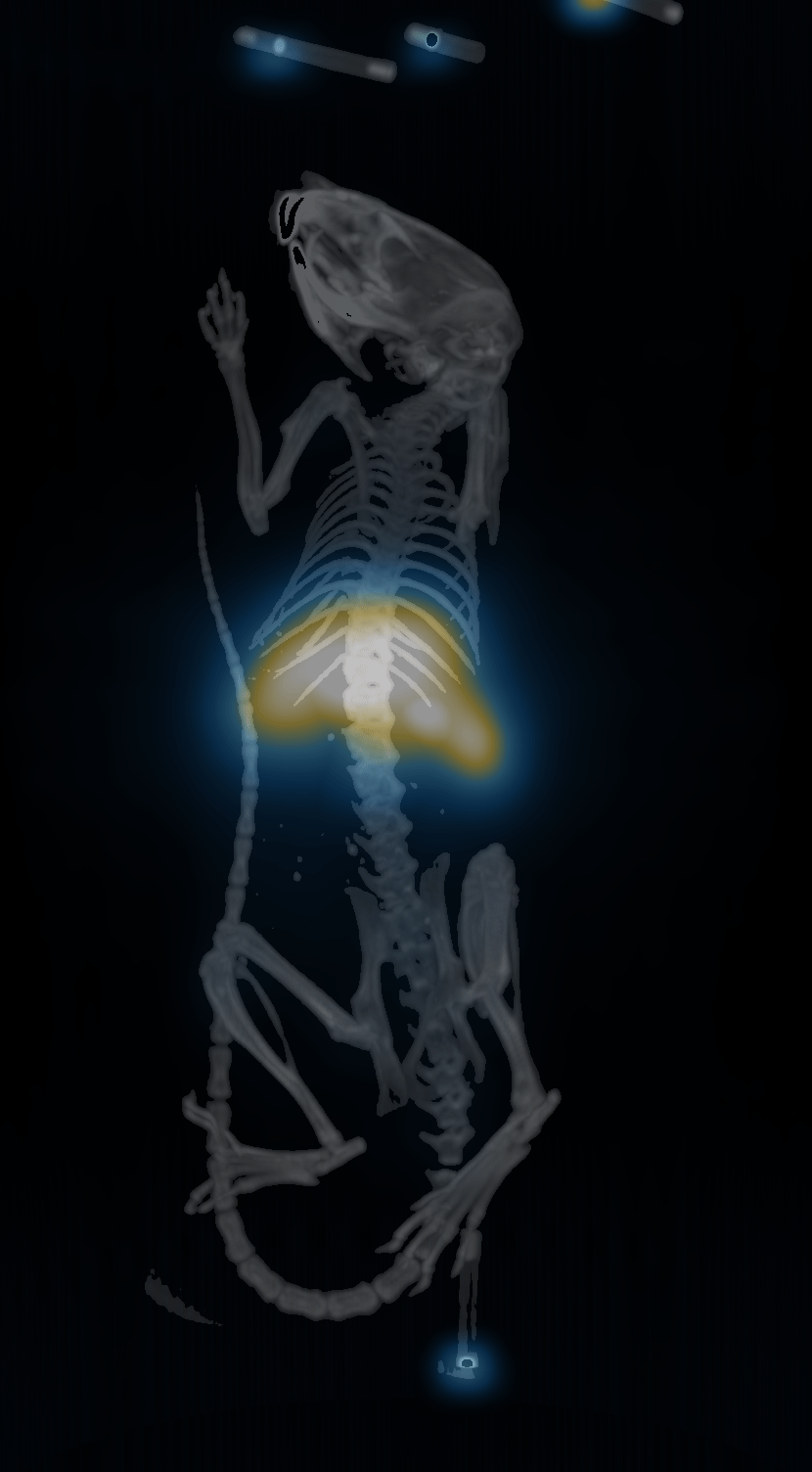

Supplement: Supplementary file 3 — Supplementary moie/video S2. [file ntnov05p0348s3.gif]

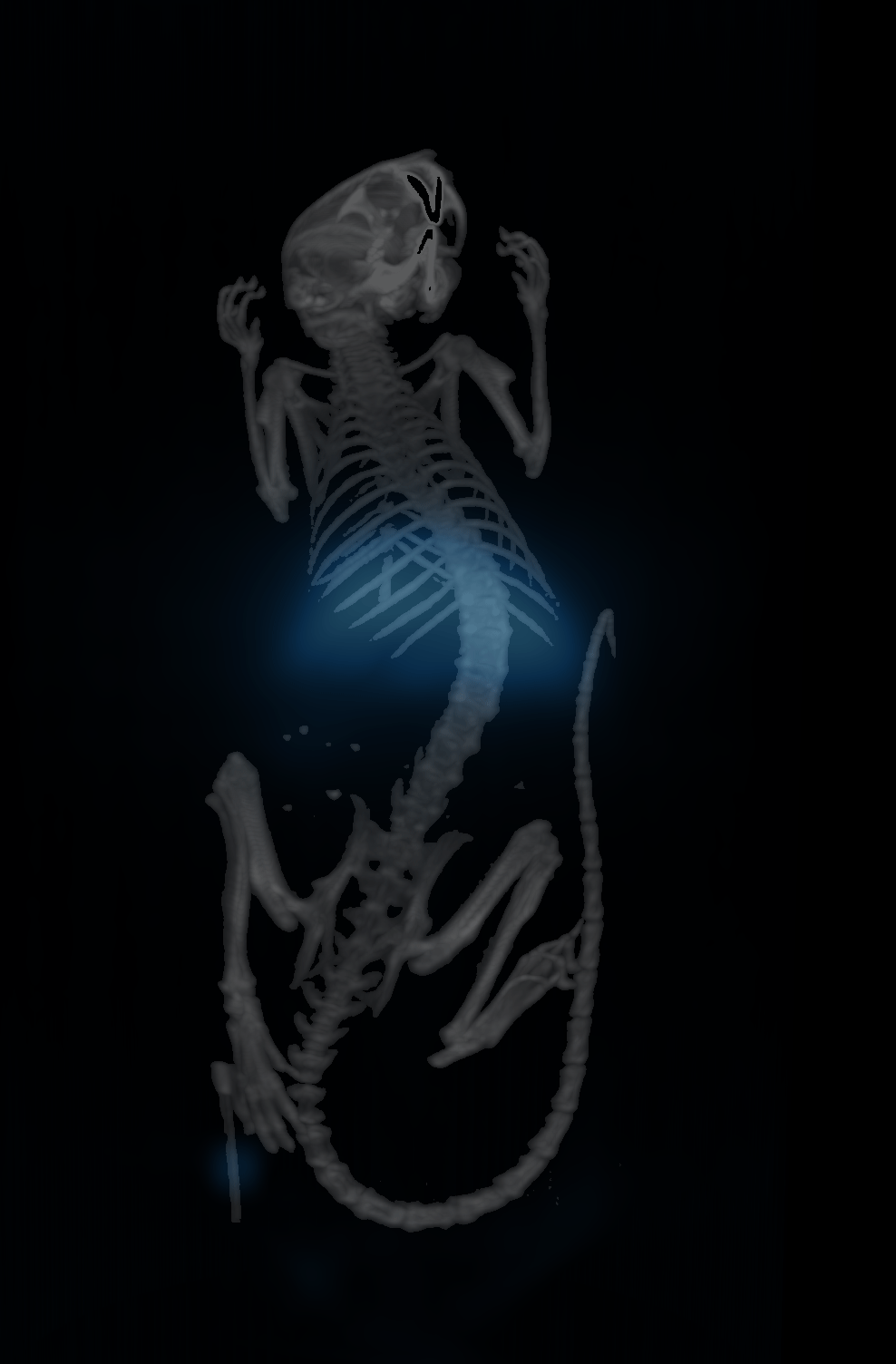

Supplement: Supplementary file 4 — Supplementary moie/video S3. [file ntnov05p0348s4.gif]

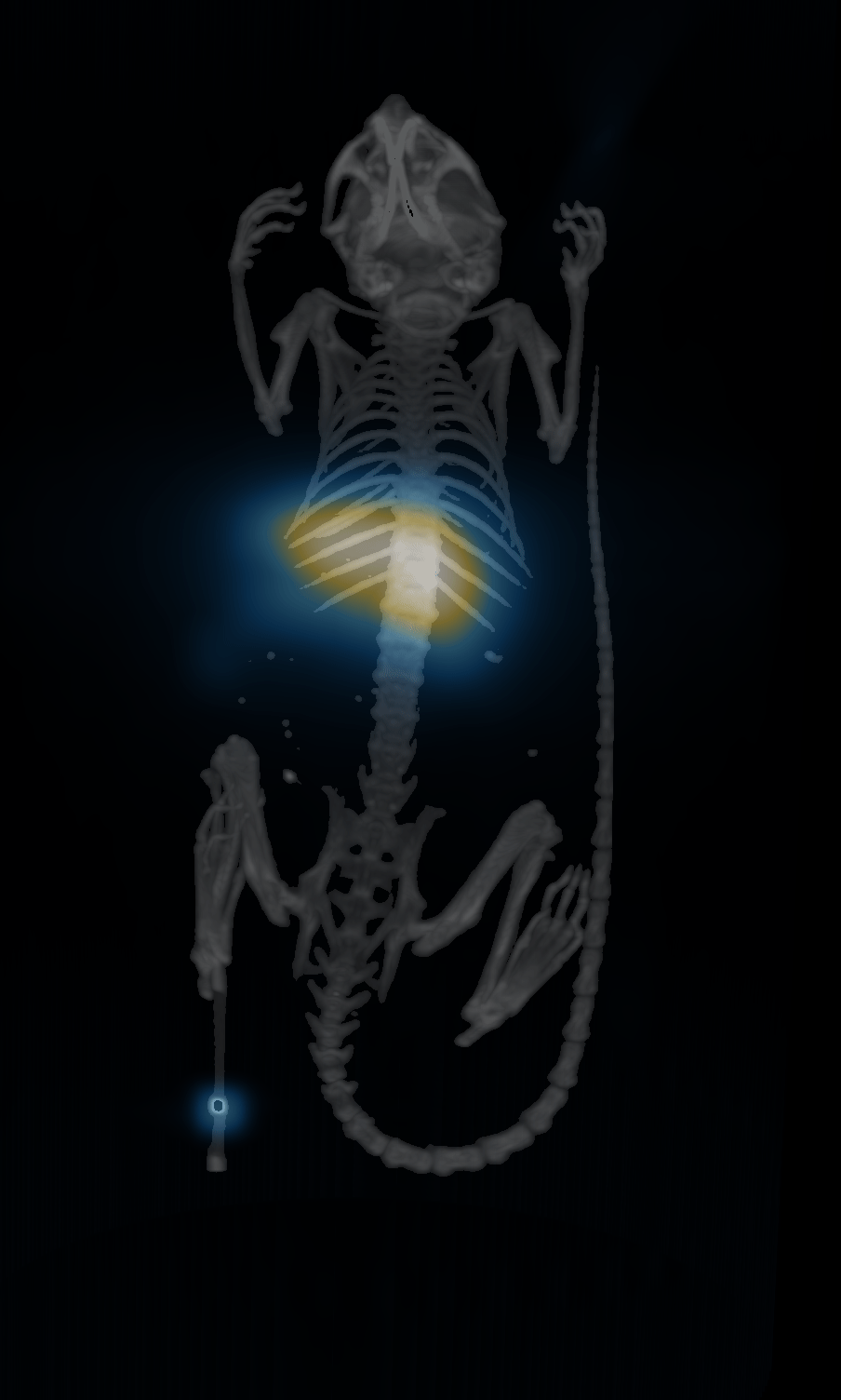

Supplement: Supplementary file 5 — Supplementary moie/video S4. [file ntnov05p0348s5.gif]
